# PAX6 promotes neuroendocrine phenotypes of prostate cancer via enhancing MET/STAT5A-mediated chromatin accessibility

**DOI:** 10.1186/s13046-024-03064-1

**Published:** 2024-05-15

**Authors:** Nan Jing, Xinxing Du, Yu Liang, ZhenKeke Tao, Shijia Bao, Huixiang Xiao, Baijun Dong, Wei-Qiang Gao, Yu-Xiang Fang

**Affiliations:** 1 State Key Laboratory of Systems Medicine for Cancer, Renji-Med-X Stem Cell Research Center, Ren Ji Hospital, School of Medicine, School of Biomedical Engineering, Shanghai Jiao Tong University, Shanghai, 200127 China; 2 Med-X Research Institutes, Shanghai Jiao Tong University, Shanghai, 200030 China; 3 Department of Urology, Ren Ji Hospital, School of Medicine, Shanghai Jiao Tong University, Shanghai, 200127 China; 4 State Key Laboratory of Molecular Developmental Biology, Institute of Genetics and Developmental Biology, Chinese Academy of Sciences, Beijing, 100101 China

**Keywords:** PAX6, Neuroendocrine prostate cancer, Lineage plasticity, STAT5A

## Abstract

**Background:**

Neuroendocrine prostate cancer (NEPC) is a lethal subset of prostate cancer which is characterized by neuroendocrine differentiation and loss of androgen receptor (AR) signaling. Growing evidence reveals that cell lineage plasticity is crucial in the failure of NEPC therapies. Although studies suggest the involvement of the neural transcription factor PAX6 in drug resistance, its specific role in NEPC remains unclear.

**Methods:**

The expression of PAX6 in NEPC was identified via bioinformatics and immunohistochemistry. CCK8 assay, colony formation assay, tumorsphere formation assay and apoptosis assay were used to illustrate the key role of PAX6 in the progression of in vitro. ChIP and Dual-luciferase reporter assays were conducted to confirm the binding sequences of AR in the promoter region of *PAX6*, as well as the binding sequences of PAX6 in the promoter regions of *STAT5A* and *MET*. For in vivo validation, the xenograft model representing NEPC subtype underwent pathological analysis to verify the significant role of PAX6 in disease progression. Complementary diagnoses were established through public clinical datasets and transcriptome sequencing of specific cell lines. ATAC-seq was used to detect the chromatin accessibility of specific cell lines.

**Results:**

PAX6 expression was significantly elevated in NEPC and negatively regulated by AR signaling. Activation of PAX6 in non-NEPC cells led to NE trans-differentiation, while knock-down of PAX6 in NEPC cells inhibited the development and progression of NEPC. Importantly, loss of AR resulted in an enhanced expression of PAX6, which reprogramed the lineage plasticity of prostate cancer cells to develop NE phenotypes through the *MET/STAT5A* signaling pathway. Through ATAC-seq, we found that a high expression level of PAX6 elicited enhanced chromatin accessibility, mainly through attenuation of H4K20me3, which typically causes chromatin silence in cancer cells.

**Conclusion:**

This study reveals a novel neural transcription factor PAX6 could drive NEPC progression and suggest that it might serve as a potential therapeutic target for the management of NEPC.

**Supplementary Information:**

The online version contains supplementary material available at 10.1186/s13046-024-03064-1.

## Introduction

In recent years, studies have shown that although the majority of prostatic tumors exhibit an androgen-driven phenotype, a considerable subset of tumors transformed to an aggressive and second-generation androgen deprivation treatment (ADT) (e.g., enzalutamide (ENZ) and abiraterone) resistant form known as neuroendocrine prostate cancer (NEPC). The NEPC exhibits characteristics of loss of androgen receptor (AR) expression, increased expression of neuronal markers, such as synaptophysin (SYP), chromogranin A (CHGA), and neuron-specific enolase (NSE, encoded by *ENO2*), is highly aggressive and lacks effective clinical interventions [1–3]. By genomic profiling studies, recurrent alterations in several key signaling pathways have been identified as potential mechanisms for neuroendocrine (NE) trans-differentiation process, including the inactivation of tumor suppressor genes such as *TP53* and *RB1* [4], the activation of the *MYCN* [5] and Aurora kinase pathways [6], and the dysregulation of the *PI3K/AKT/mTOR* pathway [7, 8]. However, Identification of additional key drivers and understanding of the related underlying molecular mechanisms for the development of NE trans-differentiation are still highly demanded so to develop novel therapeutic strategies to combat this formidable disease.

The transition from adenocarcinoma (Adeno) to NEPC is closely related to cells lineage plasticity. In fact, lineage plasticity is frequently harnessed by malignant cells to develop resistance against therapeutic interventions [9]. In this regard, prostate cancer (PCa) cells often undergo a transition towards the NE lineage after ADT, in which due to epigenetic influence, chromatin accessibility of the cells is augmented and the promoter/enhancer activity of the key driver genes for tumor progression are more active, thereby acquiring enhanced therapeutic resistance and aggressiveness [10]. NE differentiation may reflect a cell lineage transition to neural phenotypes, which mimics the neural differentiation process during embryogenesis. Addition to determinants of neuronal cell fates, many transcription factors (TFs) also show an important role in cell lineage plasticity in cancer, particularly after therapeutic treatment [11, 12]. For example, *neurogenic differentiation 1* (*NEUROD1*) which plays a crucial role in the development and differentiation of nerve cells [13], has been shown to promote the progression and metastasis of small cell lung cancer (SCLC), which has NE characteristics, by regulating the receptor tyrosine kinase B (TrkB) and neural cell adhesion molecule (NCAM) in tumor cells [14]. In addition, it has been reported that BRN2, a neurodevelopment-related TF, promotes the lineage plasticity of PCa cells and facilitates NE differentiation [12]. Therefore, Identification of novel transcription factors related to neuronal differentiation during the NEPC formation and progression would benefits our understanding of the mechanism of NEPC development.

Of many neuronal TFs, PAX6 has long been recognized as a pivotal regulator of neurogenesis in the development of the central nervous system (CNS) during embryonic development, guiding the formation of neural tube, forebrain patterning and retinal cell differentiation [15, 16]. In recent years, accumulating evidence has also shed light on the multifaceted role of *PAX6* in tumorigenesis and tumor progression, revealing its remarkable contribution to the pathological processes [17–19]. For example, *PAX6* acts as an oncogene responsible for inducing lung adenocarcinoma (LUAD) stem cell properties. The expression of *PAX6* is positively correlated with the expression of *GLI* and *SOX2*, driving cancer cells to a stem-like state [20]. However, whether or not *PAX6* plays a role during the development of NEPC has not been determined.

In this study, we compare gene expression profiling of NEPC and non-NEPC specimens, including androgen-dependent prostate cancer (ADPC) and castration-resistant prostate cancer (CRPC), and provide evidence showing that *PAX6* expression which is negatively regulated by AR signaling is elevated during the process of NE trans-differentiation. Our results suggest that *PAX6*-induced activation of the *MET/STAT5A* pathway promotes NE trans-differentiation by attenuation of H4K20me3 for the lineage switch of PCa cells towards a NE phenotype.

## Materials and methods

### Cell lines and cell culture

The human PCa cell lines LNCaP (ATCC; CRL-1740), 22Rv1(ATCC; CRL-2505), C42B (ATCC; CRL-3315), PC3 (ATCC; CRL-1435), and DU145 (ATCC; HTB-81) and human embryonic kidney 293T cell lines were obtained from the American Type Culture Collection (ATCC, Manassas, USA). 293T, 22Rv1, C42B, PC3, and DU145 cell lines were cultured in Dulbecco’s modified Eagle’s medium (DMEM; Gibco, USA) supplemented with 10% fetal bovine serum (FBS; Sigma-Aldrich, St. Louis, Missouri, USA) and 1% penicillin/streptomycin (Corning, New York, USA). LNCaP cells were cultured in RPMI-1640 medium (Gibco) supplemented with 10% FBS (Sigma-Aldrich) and 1% penicillin/streptomycin (Gibco). LNCaP^ENZ^ cell line was cultured further in the continuous presence of 20 µM ENZ (Med Chem Express, Shanghai, China) to maintain ENZ resistance. For the AR function assay, cells were maintained in androgen-depleted medium composed of phenol red-free RPMI-1640 medium, 5% charcoal/dextran-stripped serum (CSS; Gibco), and 1% penicillin/streptomycin (Gibco). All cell lines were cultured in a humidified incubator at 5%CO_2_ and 95% air atmospheres at 37℃ and were routinely tested for mycoplasma (every ~ 6 weeks) using the MycoSEQTM Mycoplasma Detection Kit (Thermos Fisher Scientific, USA). Experiments were performed using fewer than 10 passages for each cell line.

### Plasmids

A human *PAX6* lentiviral expression construct containing a puromycin resistance gene was purchased from Genomeditech (Shanghai, China). A *PAX6* P1 promoter androgen response element (ARE**)** luciferase reporter construct (*PAX6* ARE-luc) was generated by inserting the *PAX6* ARE-centric sequence, combined with a *PAX6* minimal promoter into the upstream region of the firely luciferase gene in a pGL4.17 vector (Promega, E6721). Primer sequences for cloning the *PAX6* P1 promoter sequence from LNCaP genomic DNA are provided in Supplementary Table S1.

*PAX6* short hairpin RNA (shRNA) expression constructs were purchased from Genomeditech. The *STAT5A* expression lentiviral vector was purchased from Miaoling Biology (Wuhan, China). Single-guide RNA (sgRNA) was designed using an online platform (www.benchling.com) and synthesized by Sangon Biotech Comp (Shanghai, China). The annealed DNA oligos were cloned into the pLenti-CRISPRv2 vector (Addgene_52961) for genome editing. Data from all shRNA and sgRNA sequencing methods used in this study are provided in Supplementary Table S1.

### Generation of stable knockdown and over-expression subclone cell lines

Stable *PAX6* , *STAT5A, AR, and MET* knockdown subclone cell lines were achieved by infecting cells with lentiviral vectors expressing *PAX6* shRNA (sh*PAX6*-1#, sh*PAX6*-2#), *STAT5A* shRNA (sh*STAT5A*-1#, sh*STAT5A*-2#), *AR* shRNA(sh*AR*-1#, sh*AR*-2#), and MET shRNA(sh*MET*). A non-target control shRNA was used for construction of the control subclone cell line. LNCaP and C42B cells were infected *PAX6* CDS-containing or *STAT5A* CDS-containing lentiviral vector for stably overexpressing *PAX6* or *STAT5A*. Briefly, 293T cells were co-transfected with the lentiviral vector, psPAX2 (Addgene_12260) and pMD2G (Addgene_12259) at a 3:2:1 ratio using PEI (Thermo Fisher Scientific, MD, USA) following the manufacturer’s instructions. The medium was changed 6 h after transfection. The medium containing lentivirus was harvested 48 h after transfection. PCa cells were infected with lentivirus in the presence of polybrene (8 µg/mL) followed by 2 weeks puromycin selection (5 µg/mL).

### Quantitative real-time PCR

Total RNA was extracted from the cells using the FastPure Cell/Tissue Total RNA Isolation Kit, following the manufacturer’s instructions (Vazyme, Shanghai, China). Subsequently, RNA was reverse-transcribed into cDNA using the HiScript III All-in-one RT SuperMix Perfect qPCR kit (Vazyme). qPCR was performed using the qPCR SYBR Green Master Mix (Vazyme). To ensure accuracy and reproducibility, β-actin was utilized as the internal control gene. All experimental data were obtained in triplicate and analyzed using the 2^− ΔΔCt^ method [21]. All primers used are available in Supplementary Table S1.

### Immunoblotting

Immunoblotting experiments were performed as described in our previous work [22]. Briefly, whole-cell lysates were prepared in radioimmunoprecipitation assay (RIPA) lysis buffer (Millipore, Bedford, MA, USA) supplemented with a protease inhibitor (Med Chem Express) and phosphatase inhibitor (Med Chem Express). After protein quantification using the Pierce BCA Protein Assay Kit (Thermo Fisher Scientific), 40 µg of total protein was separated via SDS-PAGE and transferred to a PVDF membrane (Millipore). The membrane was blocked with TBST containing 5% bovine serum albumin (BSA, Gibco) at 16–25 °C for 1 h and then incubated with the relevant primary antibodies at 4 °C overnight, followed by probing with a horseradish peroxidase (HRP)-conjugated secondary antibody at 16–25 °C for 1 h. The relevant proteins were visualized using an electrochemiluminescence detection instrument (Bio-Rad, California, USA) and HRP substrates. The following antibodies were used: PAX6 (Abcam, UK, ab195045), TP53(Cell Signaling Technology (CST, Danvers, MA, USA), 9282), RB1 (CST, 9313), AR (Abcam, ab133273), SYP (Proteintech, Chicago, USA, 17785-1-LG), NSE (Proteintech, 66150-1-Ap), CHGA (Proteintech, 10529-1-AP), STAT5A (CST, 94,205 and Santa Cruz Biotechnology, USA, 271,542), p-STAT5A (CST, 9359), MET (CST, 8198), p-MET (CST, 3077), Ki67 (Abcam, ab15580), KMT5C (Abclonal, Wuhan, China, A16235), and SMYD5 (Abclonal, A6191).

### Hematoxylin-eosin (H&E) and immunohistochemical (IHC) staining assays

H&E and IHC staining of paraffin-embedded tissue sections were performed by Runnerbio Biotech (Shanghai, China). Briefly, the tissues were fixed in 4% paraformaldehyde overnight and embedded in paraffin. Paraffin-embedded tissue sections (4 μm) were dewaxed in xylene for 5 min and successively hydrated in 100%, 95%, 85%, and 70% ethanol. Following inactivation of endogenous peroxidase with disodium-hydrogen phosphate-2-hydrate, these sections were blocked using 10% donkey serum for 1 h at 16–25 °C for immunohistochemical staining. Next, the sections were incubated with primary antibody (1:200) at 4 °C overnight, washed three times (10 min each time) with PBS, and then incubated with horseradish peroxidase-conjugated secondary antibody (Vector Laboratories, Burlingame, CA, USA) for 1 h at 16–25 °C. Finally, after washing three times with PBS, the sections were visualized with diaminobenzidine (DAB) staining (Sangon Biotech) and hematoxylin counterstaining (Beyotime, Shanghai, China). Images were acquired using a microscope (DFC420C; Leica, Heerbrugg, Germany).

### Immunofluorescence assay

Cells were seeded on cover slides, placed in a 24-well plate, and cultured in DMEM supplemented with 10% FBS at 5% CO_2_ at 37 °C overnight. Adherent cells on the cover slides were fixed with 4% paraformaldehyde for 15 min at 16–25 °C. The cells were blocked with 10% normal donkey serum (GeneTex, Irvine CA, USA) for 1 h at 16–25 °C. After incubation with relevant primary antibody (diluted 1:200 in PBS containing 1% normal donkey serum) at 4 °C overnight, the cells were washed for 10 min three times with PBS buffer and then incubated with Alexa Fluor-594 conjugated secondary antibody (Thermo Fisher Scientific) at 16–25 °C for 1 h in the dark. Next, the cells were washed three times with PBS and stained with DAPI (Thermo Fisher Scientific). The immunofluorescence-stained slides were observed and photographed using a microscope (Leica).

### ChIP assay

The ChIP assay was performed using a SimpleChIP Enzymatic Chromatin IP Kit (CST, 9003) according to the manufacturer’s instructions. LNCaP cells were cultured in phenol red-free medium containing 5% CSS for 72 h, after which DHT (10 nM) or DMSO was added and the cells were cultured for another 24 h. For the assay, 2 × 10^7^ cells were harvested. Briefly, chromatin was crosslinked with nuclear proteins, enzymatically digested with micrococcal nuclease, sonicated, and immunoprecipitated with anti-AR antibodies. Normal IgG included in the kit was used as the negative control for IP. Immunoprecipitates were pelleted with agarose beads, purified, and subjected to qPCR using primers specifically targeting the ARE-centric *PAX6* genomic region or the PAX6-binding *STAT5A* and *MET* promoter region. The following antibodies were used: AR (Abcam, ab108341). Flag beads (Sigma-Aldrich, M8823) were used in the PAX6 chip experiment to pull down intracellular protein-DNA conjugates after over-expression of the PAX6 plasmid in cells. The ChIP primer sequences used in this study are listed in Supplementary Table S1.

### Cell proliferation assays

To determine cell proliferation, cells were seeded on 96-well plates at a density of 2,000 cells per well and were cultured in medium with or without ENZ (20 µM, Med Chem Express) for up to 6 days. Cell proliferation was assessed using the CellTiter96 Aqueous One Solution Cell Proliferation Assay (Biosharp, Shanghai, China) according to the manufacturer’s instructions. The absorbance values of CCK-8 were measured at 450 nm using a BioTek Synergy HT microplate reader (BioTek Inc., Vermont, USA). To assess the cell growth ability after the treatment of ENZ (Med Chem Express), 2000 cells in 96-wellplate were treated with the indicated concentrations of drug and then incubated for 72 h. CCK-8 assay was performed to measure cell viability at various time points. IC50 values were calculated using Graphpad Prism.

The MTT cell proliferation assay involved seeding 2000 cells per well in a 96-well cell culture plate. After cell adhesion, 10 µL of MTT solution (Beyotime) were added to each well, and the cells are further incubated in a cell culture incubator for 4 h. Subsequently, 100 µL of formazan solution (Beyotime) was added to each well, mixed appropriately, and incubated in the cell culture incubator until the formazan dissolved completely. The absorbance was measured at 570 nm.

### Colony formation assay

For the colony formation assay, cells were seeded in 6-well plates at a density of 1,000 cells per well and cultured in medium with or without ENZ (20 µM, Med Chem Express) for up to 2 weeks. The cells were allowed to grow until visible colonies formed and were then stained with crystal violet.

### Tumorsphere formation assay

To investigate tumor sphere formation, single PCa cells were suspended in a prostate sphere culture medium consisting of DMEM/RPMI-1640 medium supplemented with N2(Gibco), B27 (Gibco), epidermal growth factor (20 ng/mL, PeproTech, New Jersey, USA), and fibroblast growth factor (20 ng/mL, PeproTech). These cells were then seeded in 24-well low-attachment dishes (Corning) at a density of 1,000 cells per well in 500 µL of medium. The culture medium was supplemented every three days until cell spheres formed, which typically occurred after approximately 1–2 weeks of culturing. The number of colonies and spheres were counted under a light microscope.

### Apoptosis assay

To detect apoptosis, cells were fixed and co-stained with propidium iodide (PI) and FITC-conjugated Annexin V using the FITC Annexin V Apoptosis Detection Kit (Yeasen, Shanghai, China), according to the manufacturer’s instructions. Briefly, 1 × 10^6^ cells were collected and incubated with Annexin V-Alexa Fluor 647 and PI for flow cytometry. The stained samples were protected from light and subjected to flow cytometry within 1 h. The independent experiment was repeated three times. Data were collected using an Accuri C6 flow cytometer and analyzed using FlowJo software (BD Biosciences Inc., New Jersey, USA).

### Luciferase reporter assay

For determining the effect of AR on *PAX6* ARE recognition, LNCaP cells were co-transfected with firefly luciferase reporter vectors containing the PAX6 ARE together with the pRL-TK renilla luciferase vector (Addgene_11313) and were treated with R1881 (1 nM, Sigma-Aldrich) combined with or without ENZ (20 µM, Med Chem Express) for 24 h. The cells were then harvested and lysed. The cell lysates were assayed for relative luciferase activity using a Dual-Luciferase Reporter Assay Kit (Yeasen) according to the manufacturer’s instructions.

### Whole transcriptome sequencing (RNA-seq) and ATAC seq

Total mRNA was reverse-transcribed into barcoded cDNA fragments using an oligo-dT primer with an attached adapter. Barcoded cDNA libraries were sequenced using an Illumina HiSeq 4000 PE150 platform (Illumina). Following quality assessment, RNA-seq reads were aligned to the reference genome (GRCh37/hg19) using HISAT2. StringTie was used to assemble and quantify the transcript abundance. DESeq2 (RRID: SCR_000154) was used to perform differential gene expression analysis of the normalized data. Three replicates for each cell line were used in the experiment. The full-gene list about gene expression profile change was shown in Supplementary Table S2.

For the ATAC-seq assay, 50,000 cells were centrifuged at 500*g* for 5 min at 4℃, and the supernatant was removed. Cells were washed once with cold PBS. Subsequently, the cells were again centrifuged at 500*g* for 5 min at 4℃ and the supernatant was removed. The cells were then suspended in cold lysis buffer. Next, the cells were again centrifuged at 500*g* for 10 min at 4℃ and the supernatant was removed. The transposition reaction system was configured using Tn5 transposase. The cell nuclear content was added to the transposing reaction system mixture, and the DNA were purified after incubation at 37℃ for 30 min. The PCR system was configured with purified DNA, and PCR amplification was performed. The final DNA libraries were run on an Illumina platform after the DNA was purified. We used an integrative genome browser (IGV) program for peak visualization. Two replicates for each cell line were used in this experiment.

### Tumor xenograft experiment

Six-week-old male nude mice (SLAC, Shanghai, China) were housed and manipulated according to the protocols approved by the Renji Hospital Medical Experimental Animal Care Commission. All animals were euthanized before 20% body weight loss occurred. All mice were maintained in a pathogen-free facility at Ren Ji Hospital. Approximately 5 × 10^6^ cells were suspended in 100 µL 50% Matrigel and injected into the right flank of nude mice. To evaluate the capacity for in vivo castration resistance, nude mice were castrated two weeks prior to subcutaneous tumor cell inoculation. ENZ (Med Chem Express, HY-70,002) 10 mg/kg or its vehicle (corn oil) was injected daily via intraperitoneal injection. The tumors were harvested, imaged, and weighed after the mice were euthanized. .

### Bioinformatic analysis

Human PCa datasets used for correlation studies or for detecting profiling changes in PAX6 among different disease subtypes were downloaded from The Cancer Genome Atlas (TCGA) database, cBioPortal database (https://www.cbioportal.org/), and Gene Expression Omnibus (GEO) datasets (GSE244024, GSE202299, GSE32967, GSE6752, GSE70380, GSE161167, GSE56288, GSE137829, GSE239593 and GSE116918, GSE21034, GSE35988, GSE3325, GSE66187, GSE40275, GSE43346, GSE16560, http://www.ncbi.nlm.nih.gov/geo/). The sequencing data from SU2C/PCF 2019 Cohort [23], Beltran 2016 Cohort [24], Gao, 2014 Cohort [25], MD Anderson 2023 Cohort [26], Fred Hutchinson 2016 Cohort [27] and Broad/Cornell 2012 Cohort [28] was downloaded from the cBioPortal database. In addition, CANCERTOOL(http://web.bioinformatics.cicbiogune.es/CANCERTOOL/index.html) and PanCanSurvPlot (https://smuonco.shinyapps.io/PanCanSurvPlot/) were used to evaluate the mRNA expression and conduct survival analysis of clinical patient samples. Correlations were determined using Pearson’s correlation coefficients. Detailed information about the analysis method of each of the datasets used was shown in Supplementary Table S3.

### Statistical analysis

All experiments were repeated at least three times and the mean and standard error (mean ± SD) values calculated. Statistically significant differences between two groups were analyzed using unpaired two-tailed Student’s t-tests, and differences between more than two groups were determined using one-way ANOVA. For all analysis, the results were considered statistically significant at *p < 0.05, **p < 0.01, and ***p < 0.001.

## Results

### PAX6 expression is significantly elevated in NEPC

**Fig. 1:**
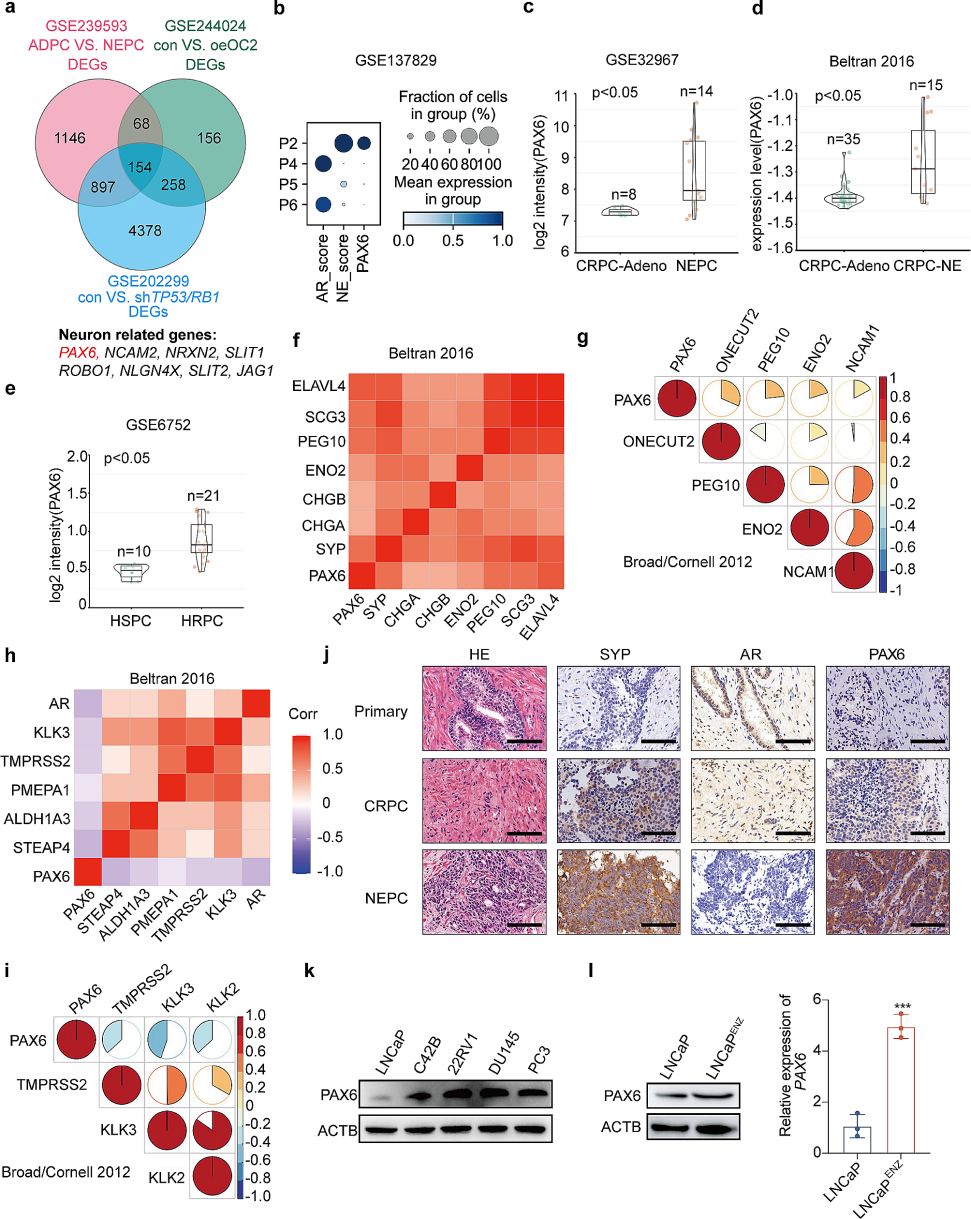
*PAX6* expression is upregulated in NEPC. **a** Intersection of differentially expressed genes from the NEPC related datasets. **b** The expression of *PAX6* in NEPC patients based on GSE137829 dataset (P2, patient 2; P4, patient 4; P5, patient 5; P6, patient 6). **c** Comparisons of *PAX6* mRNA levels level in CRPC-Adeno and NEPC based on the GSE32967 dataset (CRPC-Adeno, *n* = 8; NEPC, *n* = 14). **d** Comparisons of *PAX6* mRNA levels in CRPC-Adeno vs. CRPC-NE based on the Beltran-2016 dataset (CRPC-Adeno, *n* = 35; CRPC-NE, *n* = 15). **e** Comparisons of *PAX6* mRNA levels in HSPC vs. HRPC based on GSE6752 dataset (HSPC, *n* = 10; HRPC, *n* = 21). **f** Correlation analysis of *PAX6* with NE signature genes based on the Beltran 2016 Cohort. **g** Correlation analysis of *PAX6* with NE signature genes based on the Broad/Cornell 2012 Cohort. **h** Correlation analysis of *PAX6* with *AR* associated genes based on the Beltran 2016 Cohort. **i** Correlation analysis of *PAX6* with *AR* associated genes based on the Broad/Cornell 2012 Cohort. **j** Representative H&E and IHC staining of PAX6, AR and SYP in tissues from patients with Primary PCa, CRPC or NEPC (Scale Bar: 100 μm). **k** Protein expression of PAX6 in PCa cell lines. **l** Protein and mRNA expression of PAX6 in LNCaP^ENZ^ cells compared to the control cells. All the experiments were repeated for three times. Data represents the mean ± SD, ***p < 0.001

To screen potential candidates of NE trans-differentiation driver genes, we analyzed the expression profiling changes using data from 3 NEPC related datasets, including GSE239593 dataset (Bulk RNA-seq analysis data from a 3D-engineered PCa cell derived tissue (EPCaT) model), GSE244024 dataset (transcriptome profiling changes after over-expression of *ONECUT2* (*OC2*) in LNCaP cells) and GSE202299 dataset (transcriptome profiling changes after knockdown of *TP53* and *RB1* in C42B cells) (Fig. 1a, and Supplementary Table S3). We took the intersection of the differentially expressed genes in these three datasets (total 154 genes) and identified eight neuron-related genes (Fig. 1a). Among them, we found a novel neuron-related TF, PAX6, which exhibited an upregulation with the largest fold change in NEPC group (Fig. S1a). To confirm the above findings, we further analyzed single-cell RNA-seq data of clinical NEPC specimens from GSE137829 dataset [29] and found that *PAX6* expression was markedly high in the most advanced NEPC, accompanied with a low level of AR score (Fig. 1b).

To further study the relationship between the expression of *PAX6* and the initiation of NEPC, we performed bioinformatics assays in other public datasets and found that *PAX6* mRNA level was higher in NEPC patient-derived xenografts (PDXs) compared to Adeno PDXs (GSE32967), as well as in CRPC-NE samples compared to CRPC-Adeno samples (the Beltran 2016 Cohort [2]) (Fig. 1c and d). Consistently, analysis of a published CRPC dataset (GSE6752) also confirmed a higher expression level of *PAX6* in hormone-refractory prostate cancer (HRPC) compared to hormone-sensitive prostate cancer (HSPC) (Fig. 1e). We also studied the PAX6 expression in both human and mouse tissue samples with various AR and NE markers profiling using the data from GSE66187 dataset. We observed an upregulation of *PAX6* in AR^−^/NE^+^ NEPC-like human as well as mouse samples, which indicated a positive relationship between elevated *PAX6* expression and NEPC (Supplementary Fig. S1b and S1c). Furthermore, we observed that *PAX6* was positively correlated with the NE signature genes in the Beltran 2016 Cohort [2] (Fig. 1f) and the Broad/Cornell 2012 Cohort [28] (Fig. 1g). On the other hand, we also observed that *PAX6* was negatively correlated with *AR* associated genes such as *KLK3* in the Beltran 2016 Cohort and the Broad/Cornell 2012 Cohort (Fig. 1h and i). For further confirmation, we analyzed and verified the negative correlation between *PAX6* and *AR* expression levels in GSE32967 and GSE6752 datasets which we used above (Supplementary Fig. S1d). Next we hypothesized that high expression of PAX6 might be generalized in other neuroendocrine cancers, and we examined expression of *PAX6* in SCLC that is also a type of neuroendocrine and compared it to non-small cell lung cancer (NSCLC). We indeed observed that expression level of *PAX6* was also significantly in SCLC higher than that in NSCLC, indicating that upregulation of *PAX6* might play a general role on promotion of NE trans-differentiation in cancers (Supplementary Fig. S1e and S1f).

To validate the elevated PAX6 expression levels in NEPC tissue samples, we performed IHC and H&E staining with tissues sections prepared from human CRPC and NEPC specimens. When compared to primary PCa tissues which never received ADT treatment, PAX6 levels were indeed higher in NEPC which exhibited NE histology than in CPRC and primary PCa tissues (Fig. 1j and Supplementary Fig. S1g and Supplementary Table S4). We further wondered whether the expression of *PAX6* was also associated with other pathological characteristic such as gleason score stage and metastasis in PCa. To this end, we examined the relationship between *PAX6* expression and gleason score stage in the GSE21034 dataset [30] and found that the *PAX6* expression was markedly upregulated with the increase of gleason score (Supplementary Fig. S1h). We also found that the *PAX6* expression was significantly higher in metastatic PCa tissues than that in non-metastatic prostate carcinoma in the GSE35988 dataset [31] and the GSE3325 dataset [32] (Supplementary Fig. S1i).

Consistently, in human PCa cell lines, we found that the PAX6 expression was significantly upregulated in the DU145 and PC3 cells, with characteristics of prostatic small-cell/NE carcinoma [33] (Fig. 1k and Supplementary Fig. S1j), compared to that in the LNCaP cells, a well-known non-NEPC cell line [34]. Moreover, we examined the drug-resistant growth ability of these cells following ENZ treatment (20 µM for 6 h) and found that the proliferation ability of LNCaP cells was weakened significantly after the ENZ treatment (Supplementary Fig. S1k). Collectively, these results indicated that the expression of PAX6 was upregulated in NEPC as a response to the ENZ treatment.

Next, in order to examine the *PAX6* expressional change in ADT-induced NEPC, LNCaP cells were selected from long-time cultures in the presence of ENZ (20 µM) to construct an ENZ-resistant LNCaP subcell line named LNCaP^ENZ^, which imitated the clinical transition to NEPC under ADT [35]. We found that LNCaP^ENZ^ cells proliferated faster than parental LNCaP cells under the treatment of ENZ (Supplementary Fig. S1l), and PAX6 mRNA and protein levels were upregulated as a response to the treatment (Fig. 1l). Thus, these data together indicate that the expression of *PAX6* is positively correlated with NE trans-differentiation in PCa.

### PAX6 is necessary to maintain the NE traits and aggressive behavior of NEPC cells

**Fig. 2:**
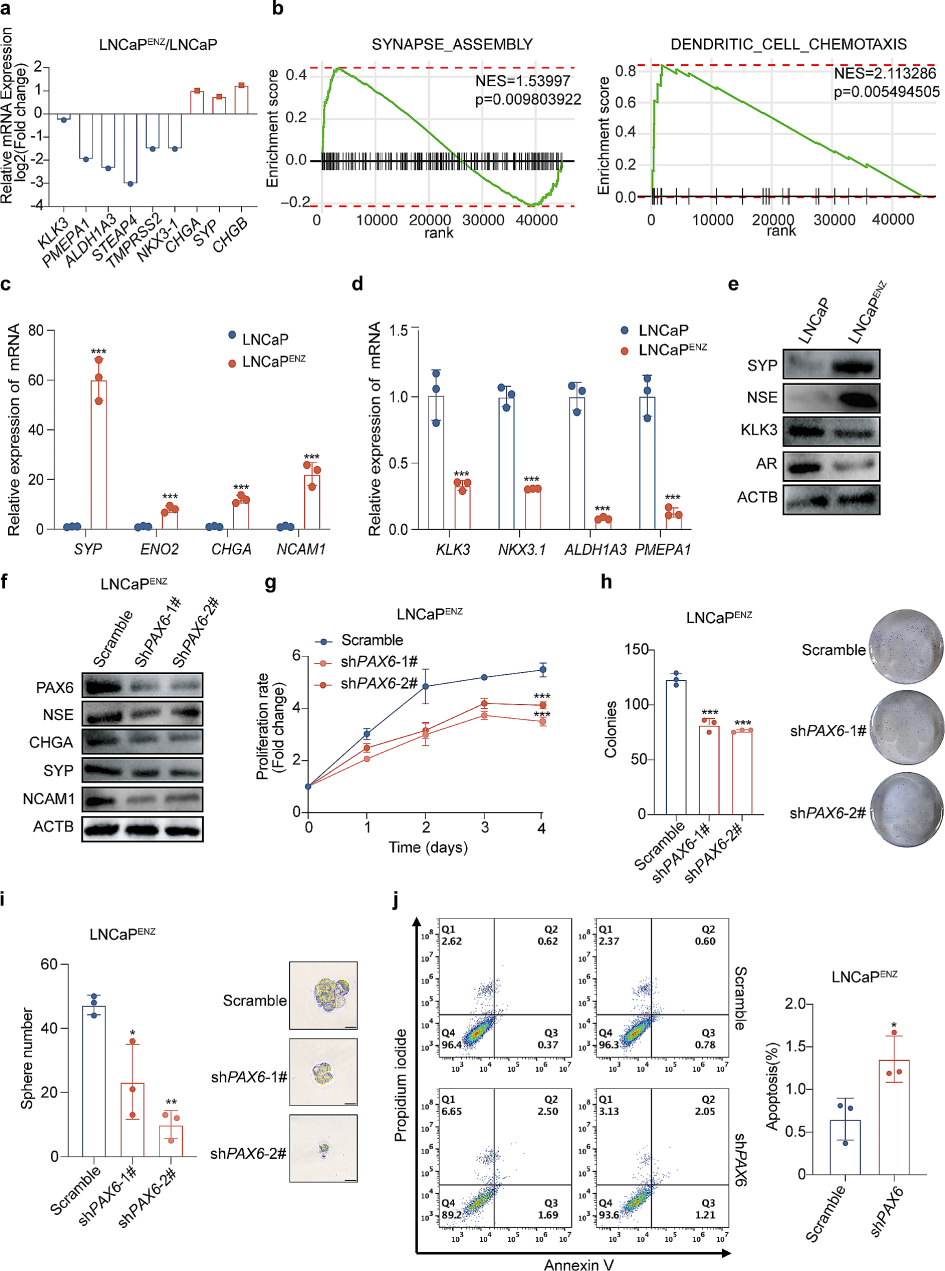
Elevated expression of *PAX6* is associated with the resistance to ENZ in PCa. **a** Relative mRNA expression of NE signature genes and *AR* associated genes in LNCaP^ENZ^ cells compared with the control by RNA-seq. **b** GSEA results of the indicated gene signatures for the comparisons of LNCaP^ENZ^ and control cells. **c** mRNA expression of NE signature genes in LNCaP^ENZ^ and control cells. **d** mRNA expression of *AR* associated genes in LNCaP^ENZ^ and control cells. **e** Protein expression of SYP, NSE, KLK3, AR in LNCaP^ENZ^ cells and control cells. **f** Protein expression of PAX6, NSE, CHGA, SYP and NCAM1 in LNCaP^ENZ^-sh*PAX6* cells and control cells. **g** Cell proliferation assays in LNCaP^ENZ^-sh*PAX6* cells and control cells. Data represent the fold change of OD value during an observation period of up to 4 days. Fold change on the day of cell seeding (day0) in each group was set as 1. **h** Representative image and quantification assay of colony numbers in LNCaP^ENZ^-sh*PAX6* cells and control cells. **i** Representative image and quantification assay of tumorsphere formation in LNCaP^ENZ^-sh*PAX6* cells and control cells. **j** Flow cytometric analysis for cell apoptosis by the percentage of Annexin V + cell population in LNCaP^ENZ^-sh*PAX6* cells and control cells. All the experiments were repeated for three times. Data represents the mean ± SD. *p < 0.05, **p < 0.01, ***p < 0.001

In order to investigate the NE signature gene profiling changes in LNCaP^ENZ^ cells, we performed transcriptome sequencing. As results shown in Fig. 2a, downregulation of *AR* associated genes (e.g. *KLK3* and *TMPRSS2*) and upregulation of NE signature genes (e.g. *CHGA*, *SYP* and *CHGB*) were observed in LNCaP^ENZ^ cells compared to the parental LNCaP cells. More importantly, we also performed Gene Set Enrichment Analysis (GSEA) with the RNA-seq data from the LNCaP^ENZ^ vs. the parental LNCaP cells and demonstrated the significant enrichment of the gene signature related to “synapse_assembly” and “dendritic_cell_chemotaxis” pathway in both of which *PAX6* played an activated role (Fig. 2b). The two pathways are reported to be associated with the NE trans-differentiation of PCa cells [36, 37] and to influence expression of NE markers, cell communication related genes and tumor microenvironment regulatory genes, contributing to the aggressive feature and poor prognosis [38–42]. In agreement with the results from the above profiling assay, we also confirmed that expression of PAX6, SYP and NSE was upregulated, and that expression of AR and KLK3 was downregulated in LNCaP^ENZ^ cells compared to the parental LNCaP cells (Fig. 2c - e).

Since LNCaP^ENZ^ cells displayed the characteristics of decreased expression of *AR* associated genes and increased expression of NE signature genes and *PAX6*, we examined whether knocking down *PAX6* in LNCaP^ENZ^ cells could restore its sensitivity to ENZ. As expected, knockdown of *PAX6* led to a decreased expression of NSE, CHGA, SYP and NCAM1 (Fig. 2f), as well as downregulated the ability of proliferation, colony and tumorsphere formation following the treatment of ENZ in LNCaP^ENZ^ cells (Fig. 2g-i). Considering NEPC cells usually have anti-apoptotic properties [43], we wondered whether *PAX6* had an effect on apoptosis of cells. To examine the effect of *PAX6* on cell apoptosis, we performed the related assay and found that the proportion of apoptosis was increased in LNCaP^ENZ^ cells after knockdown of *PAX6* (Fig. 2j). These results suggested that knockdown of *PAX6* could repress the process of NE trans-differentiation and restore the sensitivity of PCa cells to ENZ.

**Fig. 3:**
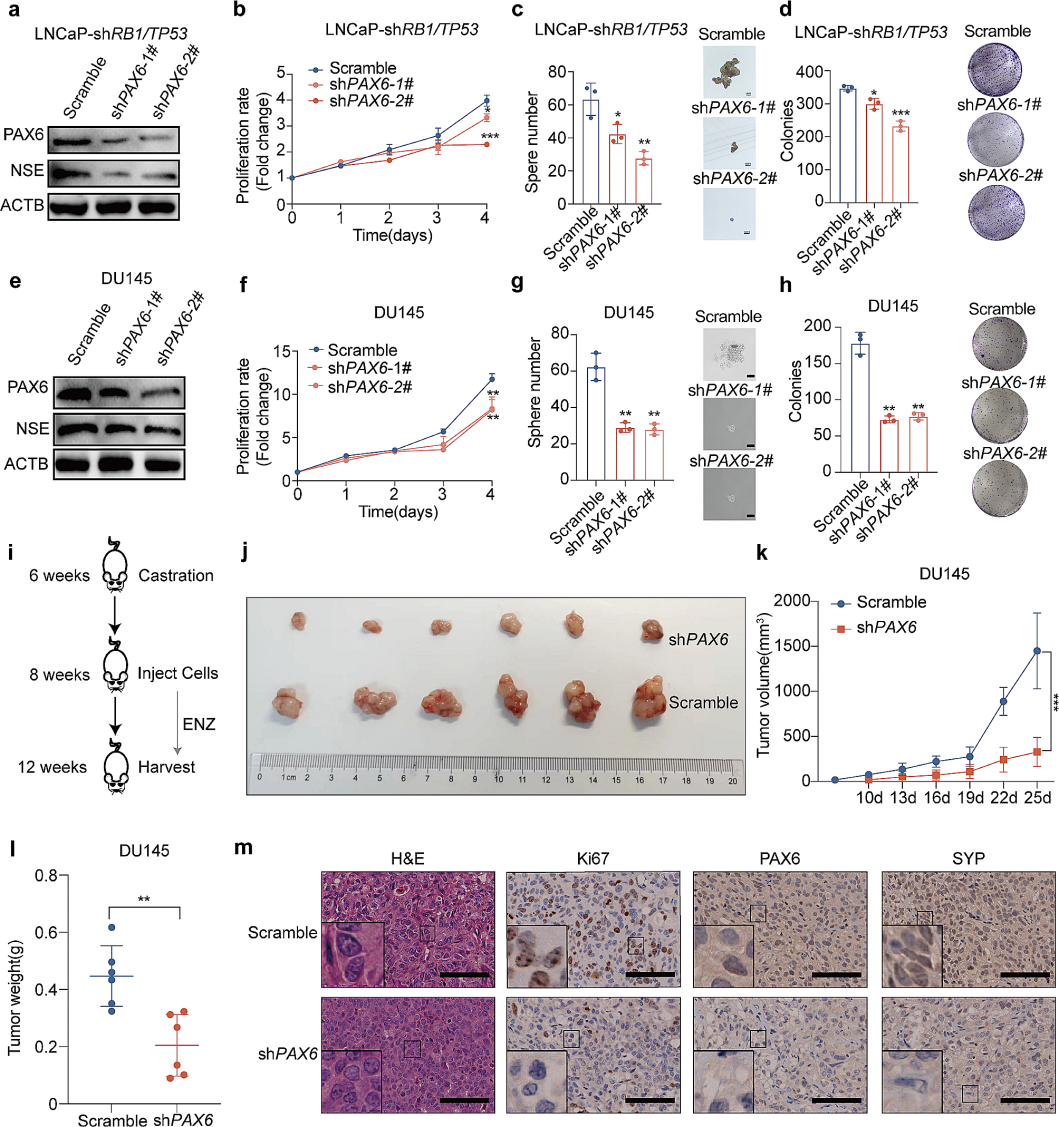
Knockdown of *PAX6* represses the phenotype of NEPC. **a** Protein expression of PAX6 and NSE after *PAX6* knockdown in LNCaP-sh*RB1/TP53* cells and control cells. **b** Cell proliferation assay after *PAX6* knockdown in LNCaP-sh*RB1/TP53* cells and control cells. **c** Representative image and quantification assay of tumorsphere formation after *PAX6* knockdown in LNCaP-sh*RB1/TP53* cells and control cells. **d** Representative image and quantification assay of colony formation after *PAX6* knockdown in LNCaP-sh*RB1/TP53* cells and control cells. **e** Protein expression of PAX6 and NSE in DU145-sh*PAX6* cells and control cells. **f** Cell proliferation assays in DU145-sh*PAX6* cells and control cells. **g** Representative image and quantification assay of tumorsphere formation after *PAX6* knockdown in DU145-sh*PAX6* cells and control cells. **h** Representative image and quantification assay of colony formation in DU145-sh*PAX6* cells and control cells. **i** Graphic of the construction of the xenograft model in castrated nude mice. **j** Anatomic tumor image of DU145-sh*PAX6* cells or control cells inoculated xenografts. **k** Tumor volume analysis of DU145-sh*PAX6* cells and control cells inoculated xenografts at the end point. **l** Tumor weight analysis of DU145-sh*PAX6* cells and control cells inoculated xenografts. **m** Representative H&E staining and IHC staining of Ki67, PAX6, SYP in xenograft samples (Scale Bar: 100 μm, with the boxed region enlarged and shown on the left). All the experiments were repeated for three times. Data represents the mean ± SD.*p < 0.05, **p < 0.01, ***p < 0.001

Previous studies have reported that loss of *TP53* and *RB1* could promote NE trans-differentiation in LNCaP cells [4]. Therefore, we constructed the LNCaP-sh*RB1/TP53* cell line as a NEPC cell model that represented more closely the clinical situations to evaluate the role of *PAX6* in regulation of NE trans-differentiation (Supplementary Fig. S2a). First, we detected an upregulation of *PAX6* expression in LNCaP-sh*RB1/TP53* cells compared to the control (Supplementary Fig. S2b). To assess the necessity of *PAX6* in maintaining the NE phenotype in PCa cells, we stably knocked down *PAX6* in the LNCaP-sh*RB1/TP53* cell line and found that the expression of NSE was downregulated compared to the control cells (Fig. 3a and Supplementary Fig. S2c). At the same time, the ability of cell proliferation, colony and tumorsphere formation was significantly reduced in the LNCaP-sh*RB1/TP53* cells under the treatment of ENZ after *PAX6* knockdown (Fig. 3b-d). Similar to what we observed in the LNCaP^ENZ^ cells, the effect of *PAX6* knockdown led to a significant increase in Annexin V^+^ cell populations, indicating an enhanced cell apoptosis at both early and late apoptotic stages in LNCaP-sh*RB1/TP53* cells (Supplementary Fig. S2d). As a further confirmation, we also downregulated *PAX6* expression in DU145 (named as DU145-sh*PAX6*, Fig. 3e and Supplementary Fig. S2e) and PC3 cells (named as PC3-sh*PAX6*, Supplementary Fig. S2h) and repeated the similar experiments described above. We found that knockdown of *PAX6* could also attenuate NE phenotypes in both two cell lines (Fig. 3f-h, and Supplementary Fig. S2f-S2k).

To verify the effect of *PAX6* on tumor growth in vivo, we performed castration on 6–8 weeks old male nude mice. Two weeks after surgery, we inoculated 5 × 10^6^ cells of DU145-sh*PAX6* or PC3-sh*PAX6* and their control cells subcutaneously and assessed the sizes and weights of tumor respectively (Fig. 3i). The results showed that the tumor volumes and weights after knockdown of *PAX6* were significantly lower than those in the control group (Fig. 3j -l and Supplementary Fig. S2l). IHC assay results showed that after knockdown *PAX6*, the expression of SYP was significantly reduced (Fig. 3m and Supplementary Fig. S2m). Collectively, we concluded that *PAX6* is essential for maintaining NE trans-differentiation and NEPC cell behaviors.

#### PAX6 promotes NE plasticity and inhibits AR signaling

**Fig. 4:**
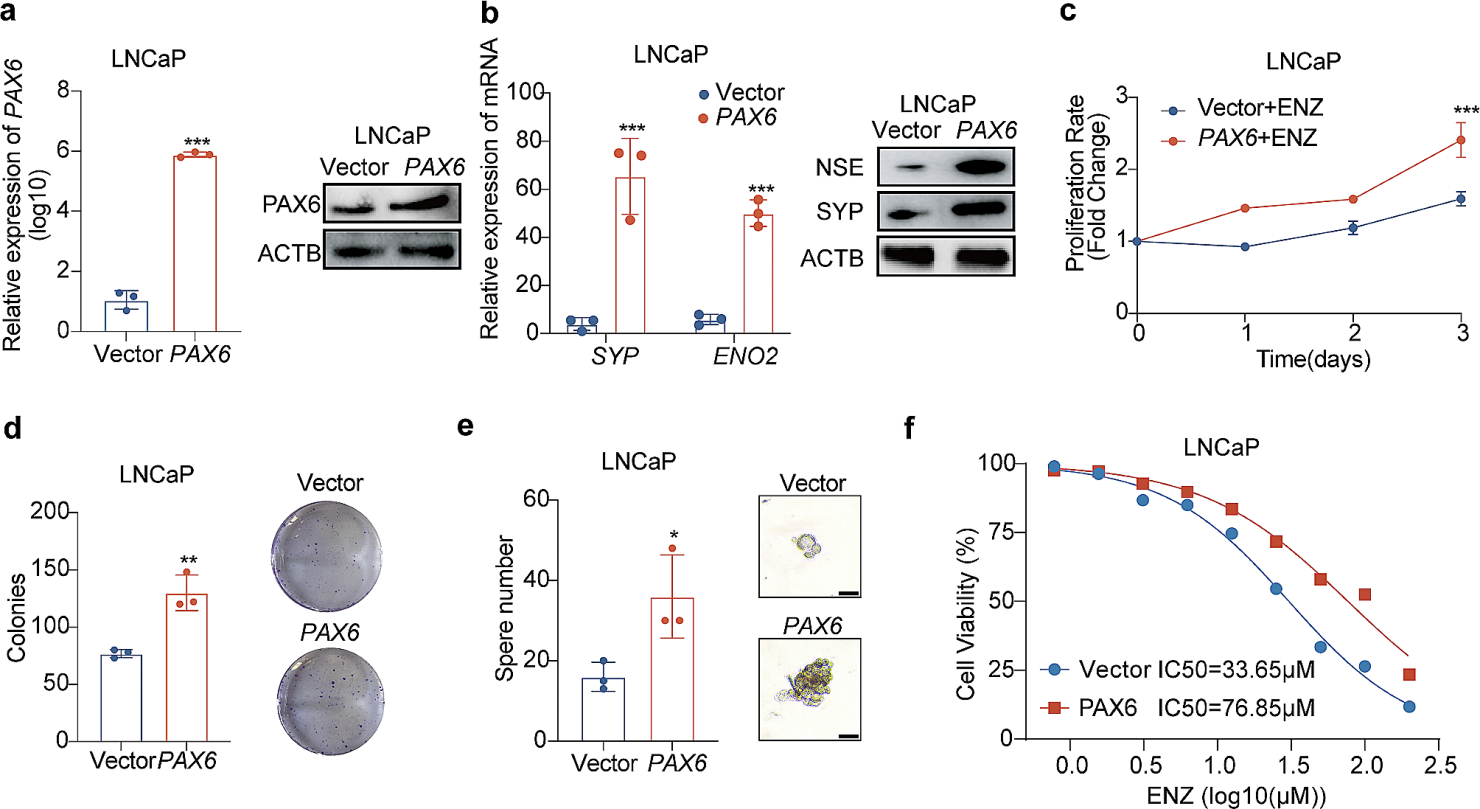
Over-expression of *PAX6* promotes the NE trans-differentiation in non-NEPC cells. **a** mRNA and protein expression of *PAX6* in LNCaP-*PAX6* cells and control cells. **b** mRNA and protein expression of *SYP* and *ENO2* genes in LNCaP-*PAX6* cells and control cells. **c** Cell proliferation assays in LNCaP-*PAX6* cells and control cells after treatment of ENZ (20 µM). **d** Representative image and quantification assay of colony number in LNCaP-*PAX6* cells and control cells. **e** Representative image and quantification assay of tumorsphere formation in LNCaP-*PAX6* cells and control cells. **f** ENZ dose–response curves for LNCaP-*PAX6* cells and control cells. All the experiments were repeated for three times. Data represents the mean ± SD. *p < 0.05, **p < 0.01, ***p < 0.001

To confirm *PAX6*’s role in induction of NE trans-differentiation in PCa cells, we stably overexpressed *PAX6* in LNCaP and C42B cells, respectively (Fig. 4a and Supplementary Fig. S3a). We found that over-expression of *PAX6* upregulated the expressional level of NE lineage markers such as SYP and NSE in LNCaP and C42B cells compared with controls (Fig. 4b and Supplementary Fig. S3b). Notably, over-expression of *PAX6* in LNCaP and C42B cells accelerated cell proliferation, colony formation and tumor sphere formation in LNCaP and C42B cells after ENZ treatment (20 µM) (Fig. 4c - e and Supplementary Fig. S3c-3e). We also found that both LNCaP-*PAX6* and control cells exhibited the dose–dependent response to the ENZ treatment, and the treatment sensitivity is lower in LNCaP-PAX6 cells (IC50: 76.85 µM) than in control cells (IC50: 33.65 µM) (Fig. 4f). As expected, we observed similar results in C42B-*PAX6* vs. control cells (Supplementary Fig. S3f). Taken together, these results suggested that *PAX6* acted as an important factor in promoting NE trans-differentiation in PCa cells.

### PAX6 is suppressed by AR activation

**Fig. 5:**
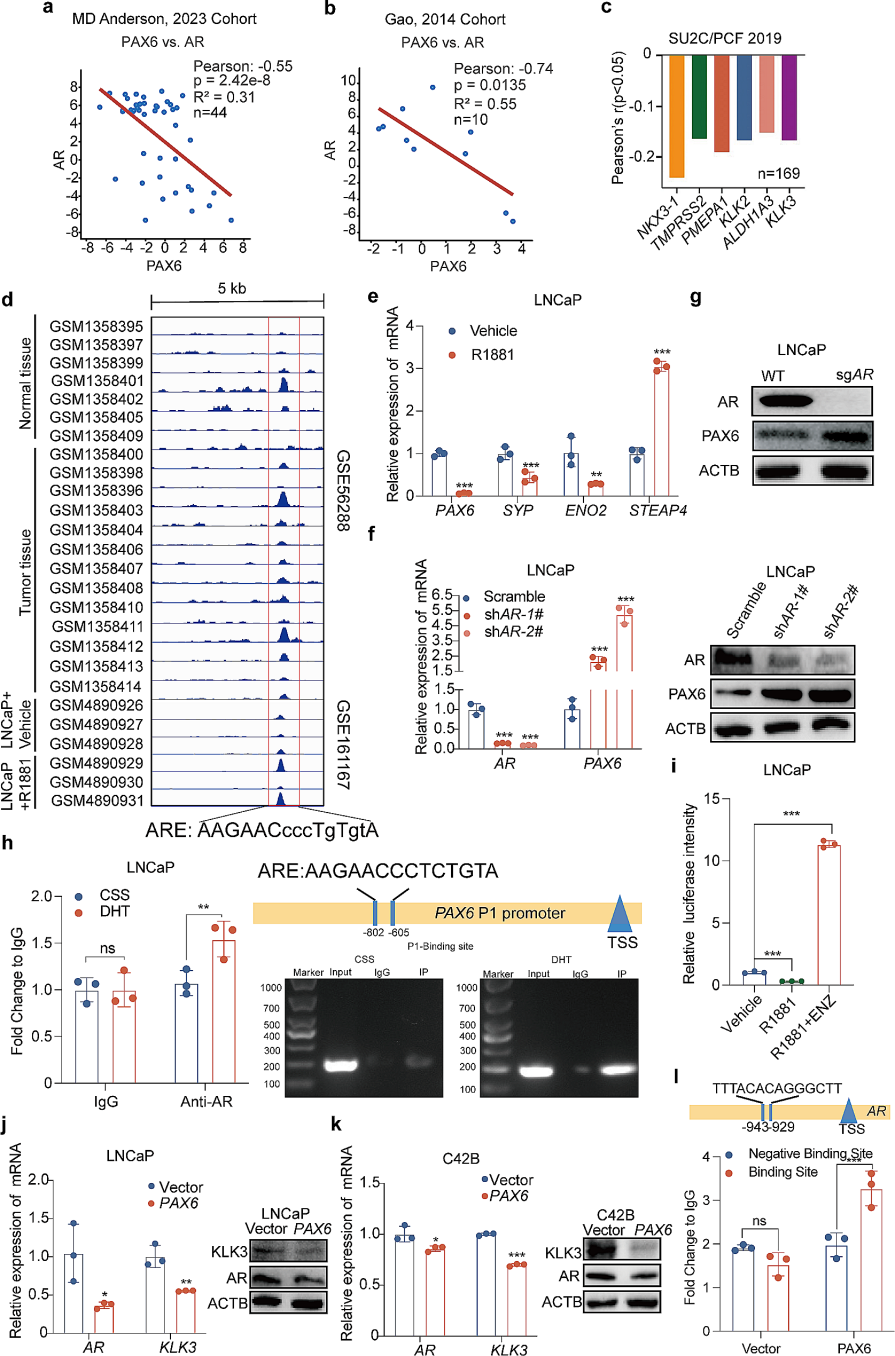
The expression of *PAX6* is negatively regulated by *AR*. **a** Correlation analysis of *PAX6* with *AR* expression based on the MD Anderson, 2023 Cohort. **b** Correlation analysis of *PAX6* with *AR* expression based on the Gao, 2014 Cohort. **c** Correlation analysis of *PAX6* with *AR* expression based on the SU2C/PCF 2019 Cohort. **d** Genomic browser representation of AR binding in *PAX6* promoter region encompassing an ARE by analysis of the data from GSE161167 (LNCaP cells) and GSE56288 (a cohort of normal and tumor human prostate tissues) datasets. **e** mRNA expression of *PAX6*, *SYP, ENO2* and *STEAP4* in LNCaP cells after treatment with R1881 (1 nM) for 6 h. **f** mRNA and protein expression of *PAX6* and *AR* after *AR* knockdown in LNCaP cells. **g** protein expression of PAX6 and AR after *AR* knockout in LNCaP cells. **h** ChIP assay of AR binding at region of the P1 promoter region of *PAX6* after treatment with DHT (10 nM) in LNCaP cells. **i** Determination of *PAX6* ARE-luc activity after treatment with R1881(1 nM, 6 h) or R1881 (1 nM, 6 h) + ENZ (20 µM, 6 h) in LNCaP cells. **j** mRNA and protein expression of *AR* and *KLK3* in LNCaP-*PAX6* and control cells. **k** mRNA and protein expression of *AR* and *KLK3* in C42B-*PAX6* and control cells. **l** ChIP assay of PAX6 binding at the promoter region of *AR.* All the experiments were repeated for three times. Data represents the mean ± SD. ns: no significance, **p < 0.01, ***p < 0.001

It has been well observed that inhibition of *AR* signaling can negatively upregulate the expression of its target genes including NE trans-differentiation related genes [44]. Therefore, we next wondered whether *PAX6* is regulated by *AR* during the process of NE trans-differentiation. We firstly detected a negative correlation of the expression of the two genes in the MD Anderson, 2023 Cohort [26] and the Gao, 2014 Cohort [25] (Fig. 5a and b). In addition, data from the SU2C/PCF 2019 Cohort [23], the Broad/Cornell 2012 Cohort [28] and the Beltran 2016 Cohort [24] revealed a negative correlation between *PAX6* and *AR* associated genes such as *NKX3-1*, *TMPRSS2*, *PMEAP1*, *KLK2*, *ALDHA13* and *KLK3* (Figs. 1h and i and 5c). Moreover, we interrogated two ChIP-seq datasets involving LNCaP cells (GSE161167) and human prostate tissues (GSE56288) and identified a consensus ARE within the *PAX6* promoter region (Fig. 5d). Thus, combining these data with our previous findings, it is plausible to suggest that *PAX6* might undergo negative transcriptional regulation by AR.

Next, we further investigated whether *AR* signaling could also negatively regulate the expression of *PAX6*. We found that after treatment with R1881(1 nM) in LNCaP cells, the *PAX6* expression was reduced along with a decreased *SYP*, *ENO2* expression and an increased *STEAP4* expression (Fig. 5e). Moreover, following steadily knockdown or knockout *AR* in LNCaP and we observed that the *PAX6* expression level was increased as a response (Fig. 5f and g). These results suggested that *PAX6* expression might be transcriptionally inhibited by AR. For further confirmation, we identified one potential ARE on the P1 promoter of *PAX6* and conducted ChIP-qPCR assay and revealed a DHT stimulation dependent binding of AR (Fig. 5h). To verify whether *AR* signaling status affects *PAX6* transcriptional activity, we incorporated the core fragment of *PAX6* promoter sequence into a luciferase reporter construct and assessed luciferase activity upon AR activation or blockade. As compared to the control, a significant decrease in luciferase activity was observed after a 6-hour treatment with R1881 in LNCaP cells. In contrast, after addition of ENZ into the culture medium as an antagonist of R1881, a restoration of luciferase activity was observed (Fig. 5i). Collectively, these results suggested that *PAX6* is transcriptionally suppressed by AR, likely via binding to an ARE in the promoter region of *PAX6*.

On the other hand, since a negative-loop feedback regulation between two genes was well-reported to be involved in the regulation of tumor progression [45, 46], we herein investigated whether PAX6 could also regulate the transcription of AR as feedback. To verify our hypothesis, we studied mRNA and protein expression of *AR* and *KLK3* in LNCaP-*PAX6* and C42B-*PAX6* cells and revealed an elevated expression of *PAX6* along with the repressive *AR* expression (Fig. 5j and k). Furthermore, we also identified a binding site of PAX6 (TTTACACAGGGCTT) in the *AR* promoter region by ChIP assay (Fig. 5l). Taken together, these results consistently indicated that there was a negative feedback regulation loop between *PAX6* and *AR*.

#### **STAT5A is a major downstream effector of PAX6 for promoting NE trans-differentiation**

**Fig. 6:**
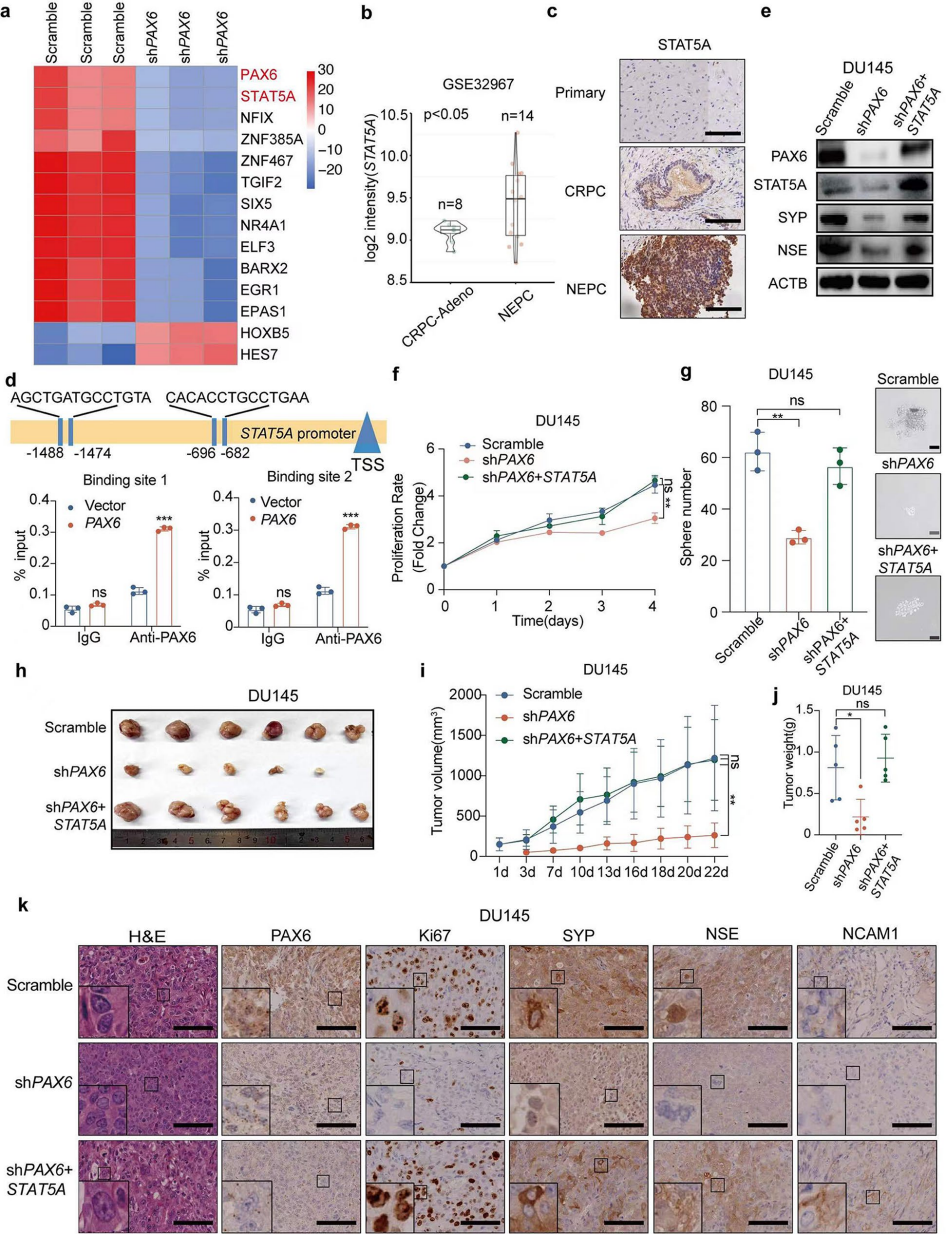
*PAX6* promotes NE characteristics via *STAT5A*. **a** The heatmap of candidate TFs with significant expressional difference in DU145-sh*PAX6* cells and DU145-Scramble cells. **b** Comparisons of *STAT5A* mRNA expression in CRPC-Adeno vs. NEPC based on the GSE32967 dataset (CRPC-Adeno, *n* = 8; NEPC, *n* = 14). **c** Representative IHC staining of STAT5A in tissues from patient with Primary PCa, CRPC or NEPC (Scale Bar: 100 μm). **d** ChIP assay of PAX6 binding at the promoter region of *STAT5A* in LNCaP-*PAX6* cells. **e** Protein expression of PAX6, STAT5A, SYP and NSE in DU145-sh*PAX6* cells with or without *STAT5A* overexpression. **f** Cell proliferation assay in DU145-sh*PAX6* cells with or without *STAT5A* overexpression. **g** Representative image and quantification assay of tumorsphere formation in DU145-sh*PAX6* cells with or without *STAT5A* over-expression. **h** Anatomic tumor images and tumor weight analysis of DU145-sh*PAX6* cells inoculated xenografts with or without *STAT5A* overexpression (*n* = 6). **i** Tumor volume analysis of DU145-Scramble, DU145-sh*PAX6* or DU145-sh*PAX6* + *STAT5A* cells inoculated xenografts respectively (*n* = 6). **j** Tumor weights analysis of DU145-sh*PAX6* and DU145-sh*PAX6* + *STAT5A* cells inoculated xenografts respectively (*n* = 6). **k** Representative staining H&E and IHC staining of PAX6, Ki67, SYP, NSE, NCAM1 in DU145-sh*PAX6* and DU145-sh*PAX6* + *STAT5A* cells inoculated xenograft samples (Scale Bar: 100 μm, with the boxed region enlarged and shown on the left, *n* = 6). All the experiments were repeated for three times. Data represents the mean ± SD. ns: no significance, *p < 0.05, ***p < 0.001

To explore potential downstream effectors of *PAX6* achieving the related aggressive behaviors in NEPC cells, we conducted RNA-seq analysis in DU145-sh*PAX6* vs. DU145-scramble cells and found that TFs exhibited certain occupancy among all of the genes with a significantly differentiated expression, which indicated that TFs might be one of the important downstream effectors in response to the knockdown of *PAX6* (Supplementary Fig. S4a). Among these TFs with significant expression differences, STAT5A was observed with a significant downregulation after knockdown of *PAX6*, which indicated that it might be a promising downstream TFs of *PAX6* to promote NE trans-differentiation (Fig. 6a).

To provide supporting evidence for the above hypothesis, we performed bioinformatics analysis and observed a positive correlation between the expression of *PAX6* and *STAT5A* in TCGA database and GSE35988 dataset [31] respectively (Supplementary Fig. S4b and S4c). Similarly, the endogenous *STAT5A* expression was also higher in samples from the NEPC patients compared to those from the CRPC-Adeno patients (Fig. 6b). More importantly, IHC assay revealed a higher expression of *STAT5A* in NEPC group than in either CRPC or primary PCa group (Fig. 6c). In addition, data from GSE70380 dataset indicated that the expression of *STAT5A* and *NCAM1* was increased following with the ENZ treatment, while the expression levels of *NKX3.1* and *KLK3* were decreased (Supplementary Fig. S4d). Moreover, we also identified a positive correlation between the expression of *STAT5A* and that of NE signature genes by analyzing the data from Broad/Cornell 2012 Cohort (Supplementary Fig. S4e). Furthermore, we analyzed data from two independent datasets (GSE16560 and GSE116918) and found that patients with high expression of the *STAT5A* had a poorer prognosis (Supplementary Fig. S4f). Therefore, these data together indicated that *STAT5A* appeared to mediate the promoting role of *PAX6* in NE trans-differentiation.

In order to confirm this possibility, we performed functional assays in vitro and in vivo to evaluate the effect of *STAT5A* expressional change on the NE trans-differentiation. First, an upregulation of STAT5A and phosphorylated STAT5A (p-STAT5A) expression was observed in LNCaP-*PAX6* cells (Supplementary Fig. S5a). We also detected a higher expression level of STAT5A and p-STAT5A in LNCaP^ENZ^ cells compared to the control cells (Supplementary Fig. S5b). Reversely, after knockdown of *PAX6* in LNCaP^ENZ^ cells, we observed a downregulation of both total STAT5A and p-STAT5A expression (Supplementary Fig. S5c). Subsequently, we investigated whether *PAX6* promoted *STAT5A* expression at a transcriptional level. To this end, we performed ChIP assay in LNCaP-*PAX6* cells and identified two binding sites of PAX6 on the *STAT5A* promoter region (Fig. 6d). Next, in order to further validate that *PAX6* induced the NE characteristics through upregulation of *STAT5A* expression, we overexpressed STAT5A in DU145-sh*PAX6* cells and PC3-sh*PAX6* cells respectively as rescue assays. We found that the expression of NE marker genes decreased with the downregulation of *PAX6* and was compensated with the over-expression of *STAT5A* in DU145 and PC3 cells (Fig. 6e and Supplementary Fig. S5d). We also found that the cell proliferation was significantly decreased following the *PAX6* knockdown but was increased to a higher or a similar level compared to that in the control after *STAT5A* overexpression in DU145 cells (Fig. 6f). In addition, we carried out tumorsphere formation assays using DU145-sh*PAX6* and PC3-sh*PAX6* cells after overexpression of *STAT5A*. We observed that the reduced sphere-forming ability due to *PAX6*-knockdown in DU145 and PC3 cells could be rescued by upregulation of *STAT5A* expression (Fig. 6g and Supplementary Fig. S5e). Reversely, knockdown of *STAT5A* in LNCaP-*PAX6* and C42B-*PAX6* cells attenuated the cell proliferation which was previously enhanced by over-expression of *PAX6* and downregulated the expression of SYP and NSE as well (Supplementary Fig. S5f and S5g).

More importantly, we expanded the above in vitro findings to an in vivo setting. After subcutaneous ectopic inoculation of DU145-sh*PAX6* or PC3-sh*PAX6* cells with or without overexpression of *STAT5A*, we found that knockdown of *PAX6* significantly inhibited tumor growth compared to the control cells, which was evidenced by the decreased tumor volume and tumor weight as well as the repressed expression of Ki67 (Fig. 6h - k and Supplementary Fig. S5h-S5k). As expected, the expression of SYP, NSE and NCAM1 was also downregulated after *PAX6* knockdown by IHC assay, which indicated that NE trans-differentiation was repressed due to the inhibition of *PAX6* expression. However, over-expression of *STAT5A* after knockdown of *PAX6* made tumor cells restore their ability of tumor growth, which exhibited no significant difference to the control cells on both tumor volume and tumor weight (Fig. 6h - k and Supplementary Fig. S5h-S5k). Consistent with the observation in vitro, the expression of SYP, NSE and NCAM1 was upregulated by overexpression of *STAT5A* as a rescue to *PAX6* knockdown (Fig. 6k and Supplementary Fig. S5k). Taken together, our findings in vitro and in vivo indicated that *PAX6* promotes NE trans-differentiation by upregulation of *STAT5A* that acts as a major effector.

#### **PAX6 induces NE trans-differentiation through the MET/STAT5A pathway**

**Fig. 7:**
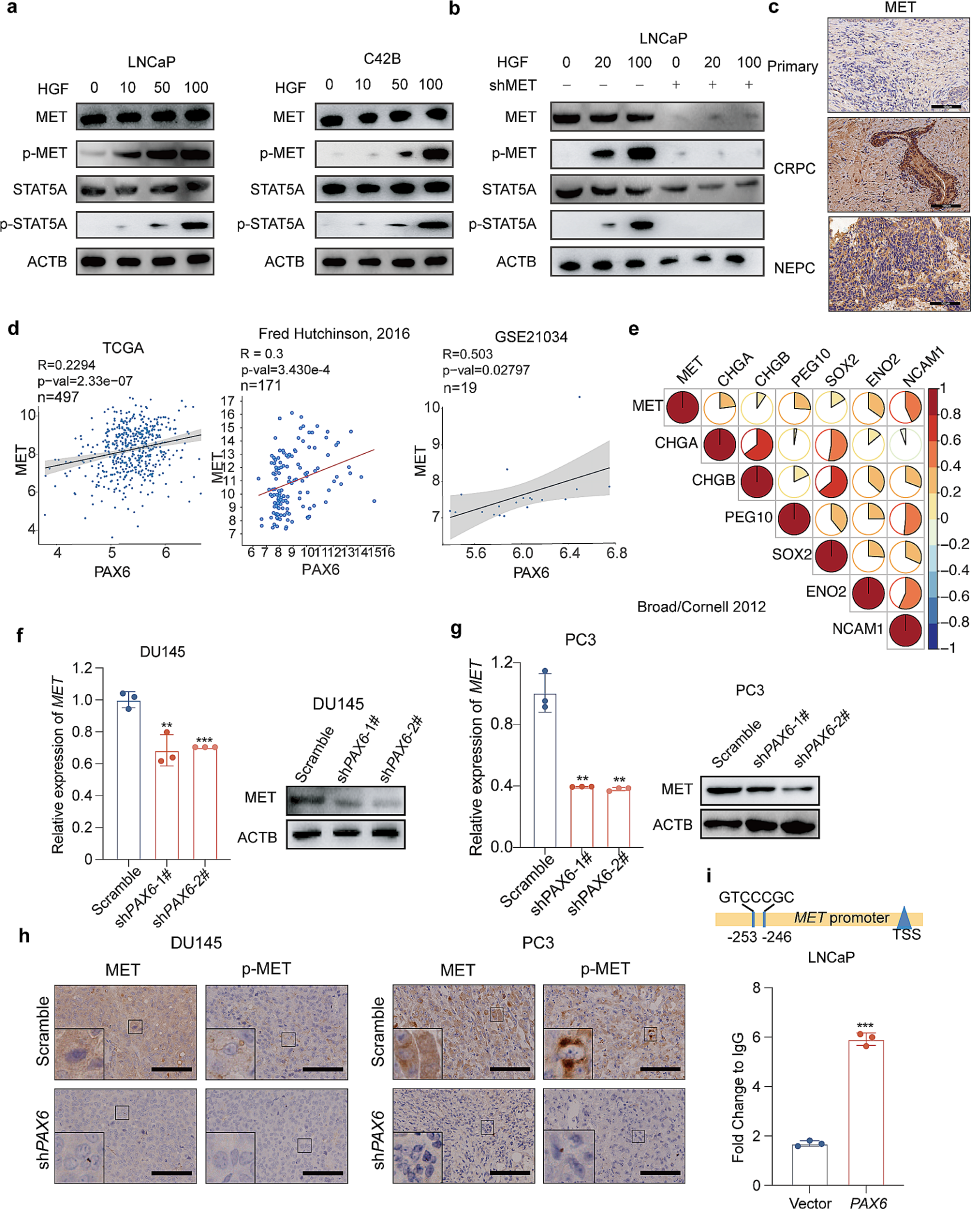
*PAX6* promotes the expression of *MET* to further phosphorylate STAT5A. **a** Protein expression of MET, p-MET, STAT5A and p-STAT5A after stimulation with different concentrations of HGF in LNCaP and C42B cells. **b** Protein expression of MET, p-MET, STAT5A and p-STAT5A after stimulation with different concentrations of HGF in *MET*-knockdown or the control LNCaP cells. **c** Representative IHC staining of MET in tissues from patients with Primary PCa, CRPC or NEPC. **d** Correlation analysis of *MET* with *PAX6* expression based on the GSE21034 dataset, TCGA database and the Fred Hutchinson, 2016 Cohort. **e** Correlation analysis of the expression of *MET* and NE signature genes based on the Broad 2012 Cohort. **f** mRNA and protein expression of *MET* in DU145-sh*PAX6* cells and control cells. **g** mRNA and protein expression of *MET* in PC3-sh*PAX6* cells and control cells. **h** Representative IHC staining of MET and p-MET in DU145-sh*PAX6* and PC3-sh*PAX6* compared with control cells inoculated xenograft samples (Scale Bar: 100 μm, with the boxed region enlarged and shown on the left). **i** ChIP assay of PAX6 binding at regions of the *MET* promoter in LNCaP cells. All the experiments were repeated for three times. Data represents the mean ± SD. ***p < 0.001

Given the fact that the STAT family members could be activated by the *MET*, a well-known receptor tyrosine kinase [47], and the elevated expression of *MET* has been reported in various cancers including PCa [48–50], we herein wondered whether phosphorylation of MET can activate STAT5A [51, 52] for promotion of NE trans-differentiation [53]. Since hepatocyte growth factor (HGF) is the sole ligand for MET [54] and is enriched in the tumor microenvironment [55], we first evaluated the function of MET on phosphorylating and activating STAT5A with the treatment of HGF in both LNCaP and C42B cells. We observed that the expression of phosphorylated MET (p-MET) was enhanced along with the increase of HGF concentration in both cells, which indicated a dose-dependent activation of MET by HGF. As a response, the phosphorylation level of STAT5A was in turn increased (Fig. 7a). Additionally, when we knocked down *MET* in LNCaP cells, STAT5A failed to be phosphorylated even under the stimulation of HGF, as a confirmation of MET mediated activation of STAT5A in PCa (Fig. 7b). At the same time, by IHC assay, we observed the elevated expression of MET in tissues from NEPC patients compared to that from either CRPC or primary PCa patients, which exhibited a similar profiling to that of PAX6 (Fig. 7c). In addition, data from the GSE116918 dataset indicated that patients with high *MET* expression showed a worse prognosis (Supplementary Fig. S6a). These results gave us a hint that the expression of *MET* might also be regulated by PAX6. To verify this possibility, we first carried out bioinformatics assays to determine the relationship between *PAX6* and *MET* expression based on the TCGA database, Fred Hutchinson, 2016 Cohort [27] and GSE21034 dataset respectively. We found that there was a positive correlation between *PAX6* and *MET* expression (Fig. 7d). In the Broad/Cornell 2012 Cohort, we also found a positive correlation between *MET* expression and the expression of NE signature genes (Fig. 7e). In both DU145-sh*PAX6* and PC3-sh*PAX6* cells, *MET* expression was downregulated at both mRNA and protein levels compared to the control (Fig. 7f and g). In sharp contrast, in both LNCaP-*PAX6* and C42B-*PAX6* cells, we observed a significant upregulation of *MET* expression after overexpression of *PAX6* (Supplementary Fig. S6b and S6c). Furthermore, we detected a significant decrease of the expression and phosphorylation levels of MET after knockdown of *PAX6* compared to the control, further supporting the notion that MET might also be a potential downstream effector of *PAX6* (Fig. 7h). To study whether PAX6 could directly bind to the *MET* promoter region and promote its transcription, we performed ChIP assay using LNCaP-*PAX6* cells, and we identified a binding site of PAX6 on the *MET* promoter region, indicating a direct regulation of the transcription of *MET* by PAX6 (Fig. 7i). Thus, our findings indicated that the elevated expression of *PAX6* promoted the expression of both MET and STAT5A as its downstream effectors to activate the *MET/STAT5A* pathway for the development of NE trans-differentiation.

#### Over-expression of PAX6 enhances cell plasticity by inhibiting H4K20me3 through STAT5A

**Fig. 8:**
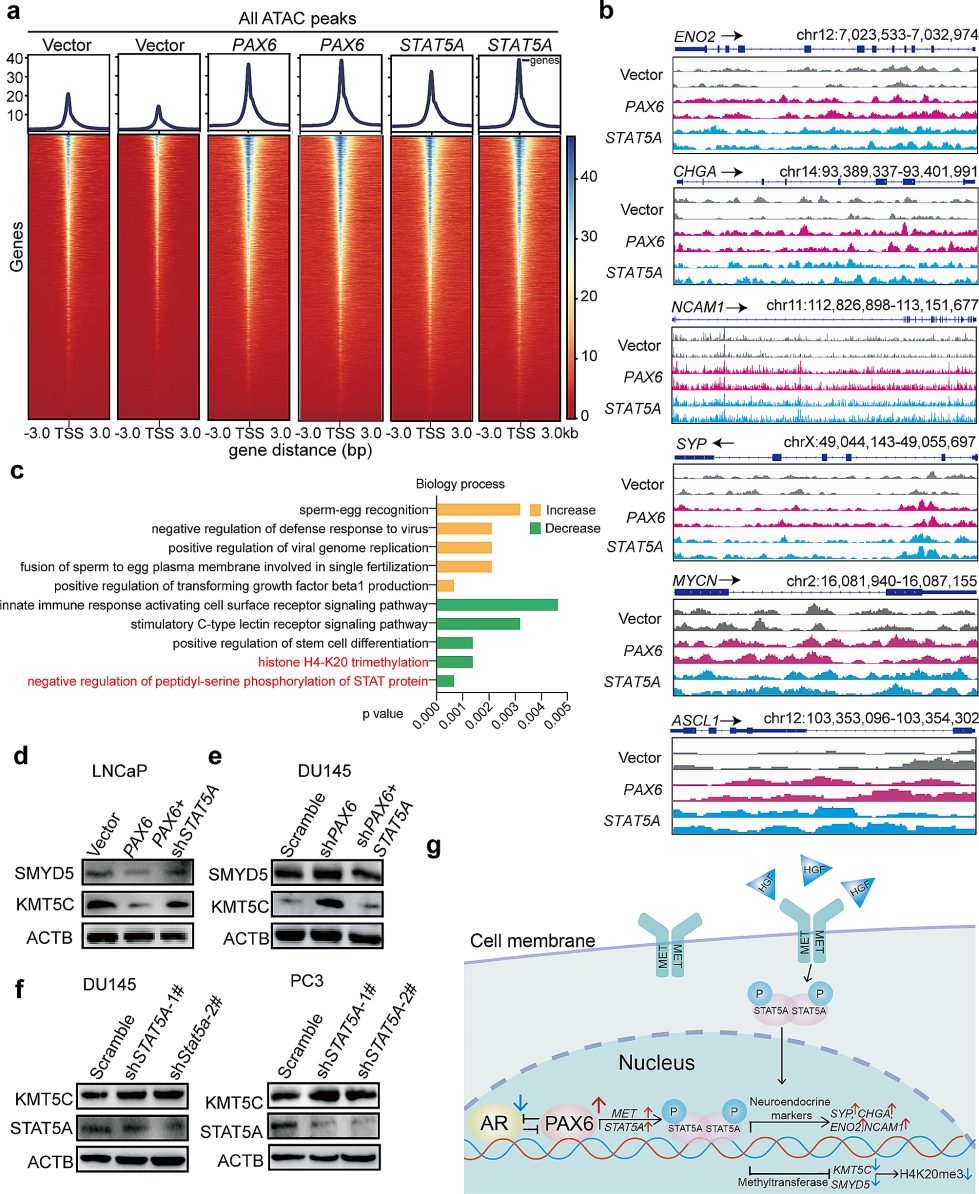
*PAX6* induced the change of lineage plasticity by attenuating the H4K20me3. **a** The heatmap showing the average ATAC-Seq signal centered on the TSS of the nearest genes in LNCaP-*PAX6*, LNCaP-*STAT5A* and control cells. **b** Chromatin accessibility of *ENO2, CHGA*, *SYP*, *NCAM1, MYCN* and *ASCL1* in LNCaP-*PAX6* or LNCaP-*STAT5A* cells compared with that in the control cells. **c** GO analysis showing the top 5 increased and decreased biological process in LNCaP-*PAX6* vs. the control cells. **d** Protein expression of SMYD5 and KMT5C in LNCaP-*PAX6* cells with or without knockdown of *STAT5A*. **e** Protein expression of SMYD5 and KMT5C in DU145-sh*PAX6* cells with or without overexpression of *STAT5A*. **f** Protein expression of KMT5C and STAT5A in DU145-sh*PAX6* and PC3-sh*PAX6* cells. **g** Graphic summary of this study

Lineage transition from Adeno to NEPC is relatively a common type of cancer cell plasticity in ADT-treated PCa [9]. It has been reported that *STAT5A* has a tightly correlation with lineage plasticity both in stem cells [56] and in tumors [57, 58]. Therefore, we wondered whether *PAX6* could promote NE trans-differentiation via *STAT5A* mediated changes of cells lineage plasticity. To this end, we performed ATAC-seq on LNCaP-*PAX6* cells or LNCaP-*STAT5A* cells to evaluate the changes of chromatin accessibility. We observed that as a response to either *PAX6* or *STAT5A* overexpression, the general chromatin accessibility was enhanced in LNCaP cells (Fig. 8a). By cluster analysis of motifs with differential accessibility, we found that both *PAX6* and *STAT5A* overexpression could enhance the chromatin accessibility and expression level of the NE markers or drivers including *SYP*, *ENO2*, *CHGA*, *NCAM1, MYCN* and *ASCL1* (Fig. 8b). Moreover, our above RNA-seq analysis in DU145-sh*PAX6* vs. Scramble cells also revealed that both synapse assembly and neurofilament bundle assembly associated genes were downregulated as a response to *PAX6* knockdown, which again indicated the *PAX6* induced profiling changes associated with the NE trans-differentiation (Supplementary Fig. S7a). Therefore, these data indicated that *PAX6*-induced activation of the *MET/STAT5A* pathway promotes NE trans-differentiation by enhancing chromatin accessibility to alter the cells lineage plasticity.

By Gene ontology (GO) enrichment analysis, we found that chromatin accessibility was increased in the region for positive regulation of TGF-beta1 production and the region for response to growth factor (Fig. 8c). Interestingly, other than these regions with increased chromatin accessibility, we also observed several decreased regions of chromatin accessibility after overexpression of *PAX6*. The top 2 significantly decreased regions of chromatin accessibility were “negative regulation of peptidyl-serine phosphorylation of STAT protein (p value < 0.03)” and “histone H4-K20 trimethylation (H4K20me3) (p value < 0.03)”, which indicated an inhibition on the process of negative regulation of STAT signaling and an attenuation of the tumor suppressive H4K20me3 after *PAX6* overexpression (Fig. 8c). These results were consistent with our above finding that *PAX6* could upregulate the expression of *STAT5A* and also gave us a hint to focus our investigation on the H4K20me3, which was an important epigenetic modification for gene silencing or repression and was well-reported to be repressed in tumors [59]. Thus, we further investigated whether elevated expression of *PAX6* could suppress H4K20me3 through activation of *STAT5A*. To this end, we detected the expression of two major methyltransferases for catalyzing the trimethylation of H4K20, KMT5C [60] and SMYD5 [61]. As expected, we observed a decreased expression of both KMT5C and SMYD5 following over-expression of PAX6 in LNCaP cells (Fig. 8d). Next, when *STAT5A* expression was inhibited in LNCaP-*PAX6* cells as a rescue assay, the expression of KMT5C and SMYD5 was increased, which indicated a negative regulation of KMT5C and SMYD5 by the *PAX6/STAT5A* axis (Fig. 8d). For further confirmation, we observed that expression of KMT5C and SMYD5 was upregulated in DU145-sh*PAX6* cells compared to the control. Moreover, when *STAT5A* was overexpressed under the condition of *PAX6* knockdown, the expression of both two genes was again repressed (Fig. 8e). In contrast, direct knockdown of *STAT5A* in either DU145 or PC3 cells significantly upregulated the expression of KMT5C and SMYD5 (Fig. 8f and Supplementary Fig. S7b) along with the downregulation of the expression of *SYP*, *ENO2*, *CHGA* (Supplementary Fig. S7c). Thus, these results together indicated that *PAX6/STAT5A* axis appears to change the lineage plasticity through inhibiting the expression of methyltransferases catalyzing the trimethylation of H4K20, such as KMT5C and SMYD5, to attenuate the H4K20me3, causing the NE trans-differentiation in PCa cells (Fig. 8g).

## Discussion

Resistance to the second-generation ADT is the main challenge for the therapy in PCa. One of the regulatory mechanisms for the resistance is the development of NE trans-differentiation for tumor progression from primary PCa to NEPC. In this study, we found that PAX6, a neuron-related TF, is selectively upregulated in ADT-induced NEPC. Activated *PAX6* signaling reprograms the chromatin accessibility via the *MET/STAT5A* axis, thereby enhancing the lineage plasticity. As a key downstream of *PAX6*, *STAT5A* inhibits the expression of two major methyltransferases KMT5C and SMYD5, both of which mediate H4k20me3. Thus, activation of the *PAX6/STAT5A* axis leads to a global downregulation of H4K20me3, triggers cancer cells lineage changing and confers a NE transcriptional profile in PCa cells. Ablation of *PAX6* in vitro and in vivo inhibits the development and progression of NEPC, and prevents the Adeno-to-NE phenotypic transition. Therefore, our study demonstrates that targeting *PAX6* is an attractive therapeutic approach for NE malignancies.

It is worth emphasizing that we have identified a novel function of *PAX6* in the regulation of NE cancer cells, which extends its role besides a coordinator of neural development in the CNS or as a key regulator of the development and maintenance of the eyes [62]. Firstly, our IHC analysis on the human NEPC samples reveals that *PAX6* is highly expressed in NEPC. Secondly, knockdown of *PAX6* in PCa cells exerts a profoundly repressive role during the progression of NEPC both in vitro and in vivo. Thirdly, in addition to PCa, we also detected a significantly higher expression of *PAX6* in another NE tumor, SCLC than NSCLC. In agreement with current findings, it has been reported that *PAX6* is critical for self-renewal of differentiation-competent radial glia-like neural stem cells [63] or acts as a transcriptional determinant in determining the transition from pluripotency to the neuroectoderm fate in human by differentially targeting pluripotent and neuroectoderm genes [16]. Finally, our sequencing results also show the enrichment of signaling pathways related to axons guidance and nerve filament development and assembly in PCa cells with a high expression of *PAX6*. The last neuronal features might be related to additional potential function in tumor metastasis or possible interactions with nerve cells or other cells such as immune cells [64, 65] in the tumor microenvironment to enhance the aggressiveness and therapy resistance, which is a subject of future studies.

Consistent with our findings, STAT family has been reported to be able to promote aggressive behavior and NE trans-differentiation in PCa cells [10, 66]. Although the *STAT5A* pathway has been well-known for promoting cell proliferation, invasion and survival in various cancers [67–69], it has not been shown whether and how this pathway is also involved in the regulation of NE trans-differentiation. It is worth mentioned that in our study, we provide several lines of evidences to demonstrate that the *MET/STAT5A* pathway works as a major downstream signaling cascade of *PAX6* for promotion of NE trans-differentiation in PCa. First, by bioinformatics and experimental assays, we revealed a positive correlation between the expression of *PAX6* and the expression of *STAT5A* or *MET*. Second, overexpression of *STAT5A* upregulates the expression of NE signature genes such as *SYP, CHGA, ENO2* and *NCAM1*. Third, by rescue assays in vitro and in vivo, knockdown of *STAT5A* reverses the phenotype of NE trans-differentiation in PCa, even under the condition of *PAX6* overexpression. Fourth, activation of the *PAX6/STAT5A* axis can change the lineage plasticity mainly by attenuation of H4K20me3 modification. Supporting for our findings comes from a previous report showing STAT3 as a key regulator of lineage plasticity to enhance the chromatin accessibility to promote NE trans-differentiation in PCa, during which *STAT3* expression can be induced by multiple upstream TFs such as *YIN YANG* 1 [70]. Therefore, the current study adds a sub-member of STAT family, STAT5A, which attenuates H4K20me3 in PCa, as a new molecule to the list that regulates the lineage plasticity.

Moreover, our study uncovers altered epigenetic modulation of histone, which acts to orchestrate the Adeno-to-NE lineage transition. Due to the requirement of massive gene expressional changes during the lineage shift, epigenetic alteration has been proposed to be actively involved. However, the upstream signals and regulators that trigger the epigenetic reprogramming remain to be identified. Nevertheless, our results demonstrate that H4K20me3 is attenuated by the *PAX6/STAT5A* axis-induced inhibition of methyltransferases KMT5C and SMYD5 in PCa cells. Using ATAC-seq assay, we uncover that the *PAX6* and *STAT5A* activation leads to a global change in transcriptional output, in particular, an increased NE lineage attribution, including enhanced expression of neuron-related genes such as *SYP, CHGA, ENO2*, *NCAM1*, axon guidance associated genes, synapse assembly and neurofilament bundle assembly associated genes.

In summary, our study demonstrates that elevated expression of *PAX6* changes the lineage plasticity to promote NE trans-differentiation via activation of the downstream *MET/STAT5A* pathway. Although ADT-induced NEPC is a category of highly aggressive malignancies with an extremely poor prognosis and a lack of effective targeted therapies, our findings indicate that attenuation of *PAX6* function or inhibiting its expression might be a potential therapeutic strategy to restore the sensitivity to the second-generation ADT in NEPC.

### Electronic supplementary material

Below is the link to the electronic supplementary material.

Supplementary Material 1

Supplementary Material 2

Supplementary Material 3

Supplementary Material 4

Supplementary Material 5

Supplementary Material 6

Supplementary Material 7

## Data Availability

RNA-seq and ATAC-seq data in this study is available in GEO database (GSE250422). Other data that support the findings of this study are available from the corresponding author upon reasonable request.

## Abbreviations

Adeno: Adenocarcinoma
ADPC: Androgen-dependent prostate cancer
ADT: Androgen deprivation treatment
AR: Androgen receptor
ARE: Androgen response element
BCR: Biochemical recurrence
CNS: Central nervous system
CRPC: Castration-resistant prostate cancer
DFS: Disease free survival
ENZ: Enzalutamide
GEO: Gene Expression Omnibus
GSEA: Gene Set Enrichment Analysis
HRPC: Hormone-refractory cancers
HSPC: Hormone-sensitive cancers
H4K20me3: Histone H4-K20 trimethylation
IHC: Immunohistochemical
LUAD: Lung adenocarcinoma
MFS: Metastasis-free survival
NE: Neuroendocrine
NEPC: Neuroendocrine prostate cancer
NSCLC: Non-neuroendocrine small cell lung cancer
OS: Overall survival
PAX6: Paired box 6
PCa: Prostate cancer
PDX: Patient-derived xenograft
SCLC: Small cell lung cancer
TF: Transcription factor
TCGA: The Cancer Genome Atlas
