# Correction: PAX6 promotes neuroendocrine phenotypes of prostate cancer via enhancing MET/STAT5Amediated chromatin accessibility

**DOI:** 10.1186/s13046-024-03084-x

**Published:** 2024-06-15

**Authors:** Nan Jing, Xinxing Du, Yu Liang, ZhenKeke Tao, Shijia Bao, Huixiang Xiao, Baijun Dong, Wei-Qiang Gao, Yu-Xiang Fang

**Affiliations:** 1 State Key Laboratory of Systems Medicine for Cancer, Renji-Med-X Stem Cell Research Center, Ren Ji Hospital, School of Medicine, School of Biomedical Engineering, Shanghai Jiao Tong University, Shanghai, 200127 China; 2 Med-X Research Institutes, Shanghai Jiao Tong University, Shanghai, 200030 China; 3 Department of Urology, Ren Ji Hospital, School of Medicine, Shanghai Jiao Tong University, Shanghai, 200127 China; 4 State Key Laboratory of Molecular Developmental Biology, Institute of Genetics and Developmental Biology, Chinese Academy of Sciences, Beijing, 100101 China

**Correction: J Exp Clin Cancer Res 43, 144 (2024)**

10.1186/s13046-024-03064-1

Following publication of the original article [1], an incorrect spelling was spotted in Fig. 6h of the published article. The tumor sample grouping as “PAX6 + shSTAT5A” should be corrected to “shPAX6 + STAT5A”.

**Fig. 6:**
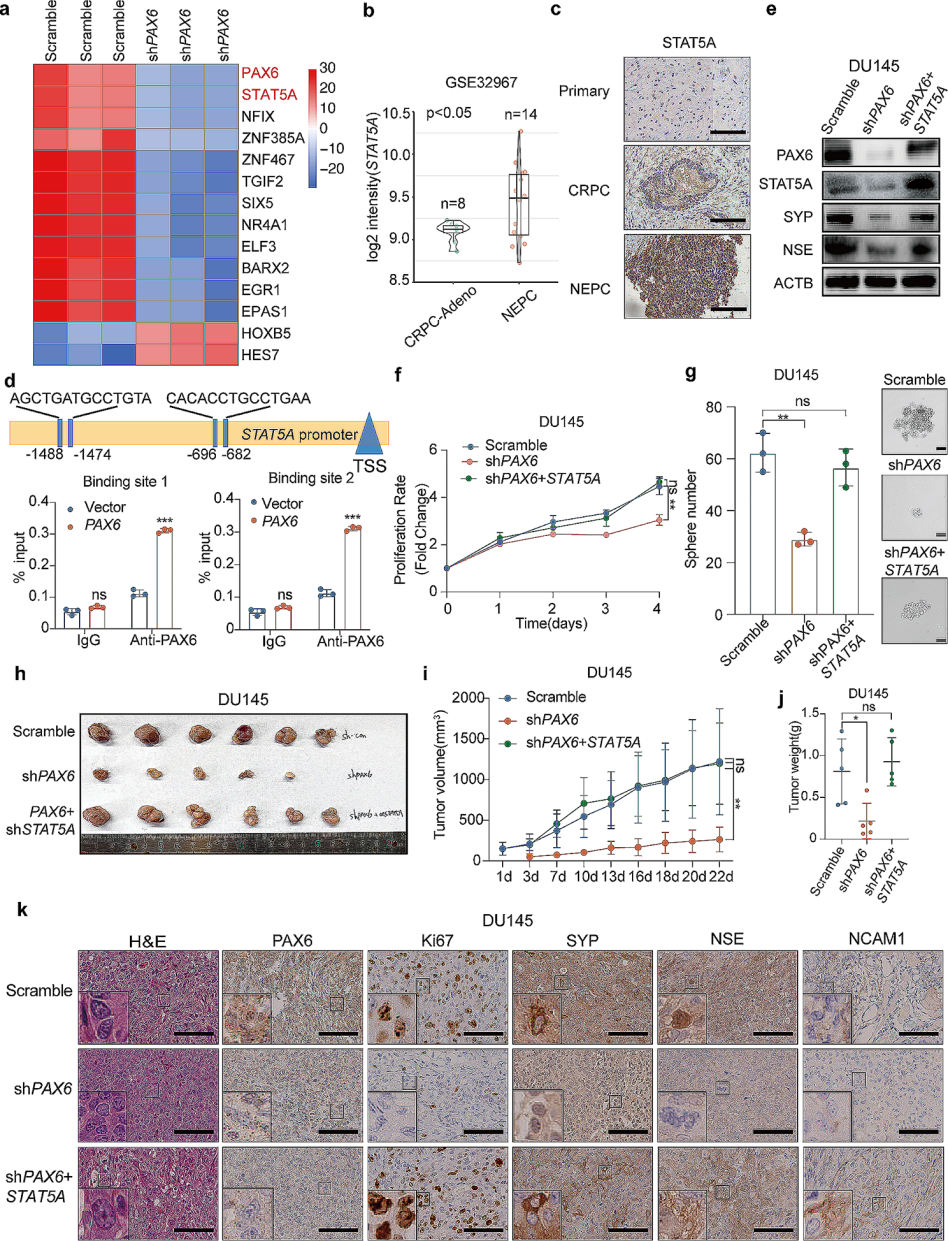
*PAX6* promotes NE characteristics via *STAT5A*. **a** The heatmap of candidate TFs with significant expressional difference in DU145-sh*PAX6* cells and DU145-Scramble cells. **b** Comparisons of *STAT5A* mRNA expression in CRPC-Adeno vs. NEPC based on the GSE32967 dataset (CRPC-Adeno, *n* = 8; NEPC, *n* = 14). **c** Representative IHC staining of STAT5A in tissues from patient with Primary PCa, CRPC or NEPC (Scale Bar: 100 μm). **d** ChIP assay of PAX6 binding at the promoter region of *STAT5A* in LNCaP-*PAX6* cells. **e** Protein expression of PAX6, STAT5A, SYP and NSE in DU145-sh*PAX6* cells with or without *STAT5A* overexpression. **f** Cell proliferation assay in DU145-sh*PAX6* cells with or without *STAT5A* overexpression. **g** Representative image and quantification assay of tumorsphere formation in DU145-sh*PAX6* cells with or without *STAT5A* over-expression. **h** Anatomic tumor images and tumor weight analysis of DU145-sh*PAX6* cells inoculated xenografts with or without *STAT5A* overexpression (*n* = 6). **i** Tumor volume analysis of DU145-Scramble, DU145-sh*PAX6* or DU145-sh*PAX6* + *STAT5A* cells inoculated xenografts respectively (*n* = 6). **j** Tumor weights analysis of DU145-sh*PAX6* and DU145-sh*PAX6* + *STAT5A* cells inoculated xenografts respectively (*n* = 6). **k** Representative staining H&E and IHC staining of PAX6, Ki67, SYP, NSE, NCAM1 in DU145-sh*PAX6* and DU145- sh*PAX6* + *STAT5A* cells inoculated xenograft samples (Scale Bar: 100 μm, with the boxed region enlarged and shown on the left, *n* = 6). All the experiments were repeated for three times. Data represents the mean ± SD. ns: no significance, *p < 0.05, ***p < 0.001

**Fig. 6:**
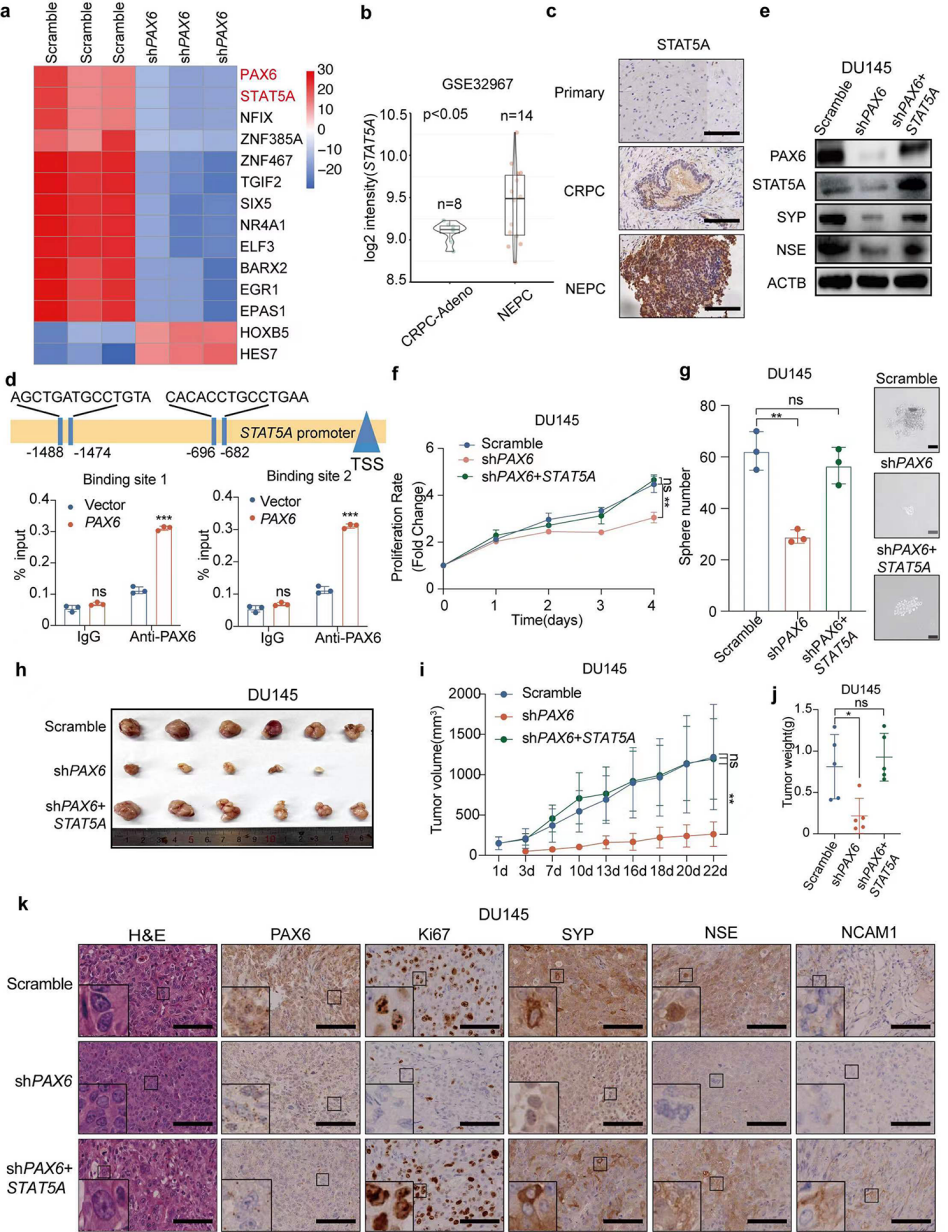
*PAX6* promotes NE characteristics via *STAT5A*. **a** The heatmap of candidate TFs with significant expressional difference in DU145-sh*PAX6* cells and DU145-Scramble cells. **b** Comparisons of *STAT5A* mRNA expression in CRPC-Adeno vs. NEPC based on the GSE32967 dataset (CRPC-Adeno, *n* = 8; NEPC, *n* = 14). **c** Representative IHC staining of STAT5A in tissues from patient with Primary PCa, CRPC or NEPC (Scale Bar: 100 μm). **d** ChIP assay of PAX6 binding at the promoter region of *STAT5A* in LNCaP-*PAX6* cells. **e** Protein expression of PAX6, STAT5A, SYP and NSE in DU145-sh*PAX6* cells with or without *STAT5A* overexpression. **f** Cell proliferation assay in DU145-sh*PAX6* cells with or without *STAT5A* overexpression. **g** Representative image and quantification assay of tumorsphere formation in DU145-sh*PAX6* cells with or without *STAT5A* over-expression. **h** Anatomic tumor images and tumor weight analysis of DU145-sh*PAX6* cells inoculated xenografts with or without *STAT5A* overexpression (*n* = 6). **i** Tumor volume analysis of DU145-Scramble, DU145-sh*PAX6* or DU145-sh*PAX6* + *STAT5A* cells inoculated xenografts respectively (*n* = 6). **j** Tumor weights analysis of DU145-sh*PAX6* and DU145-sh*PAX6* + *STAT5A* cells inoculated xenografts respectively (*n* = 6). **k** Representative staining H&E and IHC staining of PAX6, Ki67, SYP, NSE, NCAM1 in DU145-sh*PAX6* and DU145- sh*PAX6* + *STAT5A* cells inoculated xenograft samples (Scale Bar: 100 μm, with the boxed region enlarged and shown on the left, *n* = 6). All the experiments were repeated for three times. Data represents the mean ± SD. ns: no significance, *p < 0.05, ***p < 0.001

Furthermore, errors were also spotted in the Supplementary Materials:

1. Supplement material 1: In the a-panel of Figure S3, the authors omitted to mark the significant difference of the qPCR result and now have added it according to the description in the text.
2. The “Additional information” file of this paper should be deleted because it is not the final version of the supporting data.
3. Supplement material 1: In the h-panel of Figure S5, the authors misspelled the name of the tumor sample grouping as “PAX6 + shSTAT5A”. The correct spelling should be “shPAX6 + STAT5A”.
4. Supplementary material 6 should be deleted because it is the response figure which is included in our response to the reviewers’ comments for argument and is also partially repeated to the supplementary material 1.
5. Supplementary material 7 should be deleted because it is actually the supplementary figure S7 and its relevant figure legend which have been included in the supplementary material 1.

The corrections do not affect the overall result or conclusion of the article. The original article has been corrected.

Incorrect Figure 6

Correct Figure 6

### Electronic supplementary material

Below is the link to the electronic supplementary material.

Supplementary Material 1
