# Supplementary material for: PAX6 promotes neuroendocrine phenotypes of prostate cancer via enhancing MET/STAT5A-mediated chromatin accessibility
Source: J Exp Clin Cancer Res. 2024 May 15;43:144. doi: 10.1186/s13046-024-03064-1 (PMC11094950; doi:10.1186/s13046-024-03064-1)
Supplement: Supplementary file 1 — Supplementary Material 1 [file 13046_2024_3064_MOESM1_ESM.docx]

**Supplemental Figures and Figure legends**


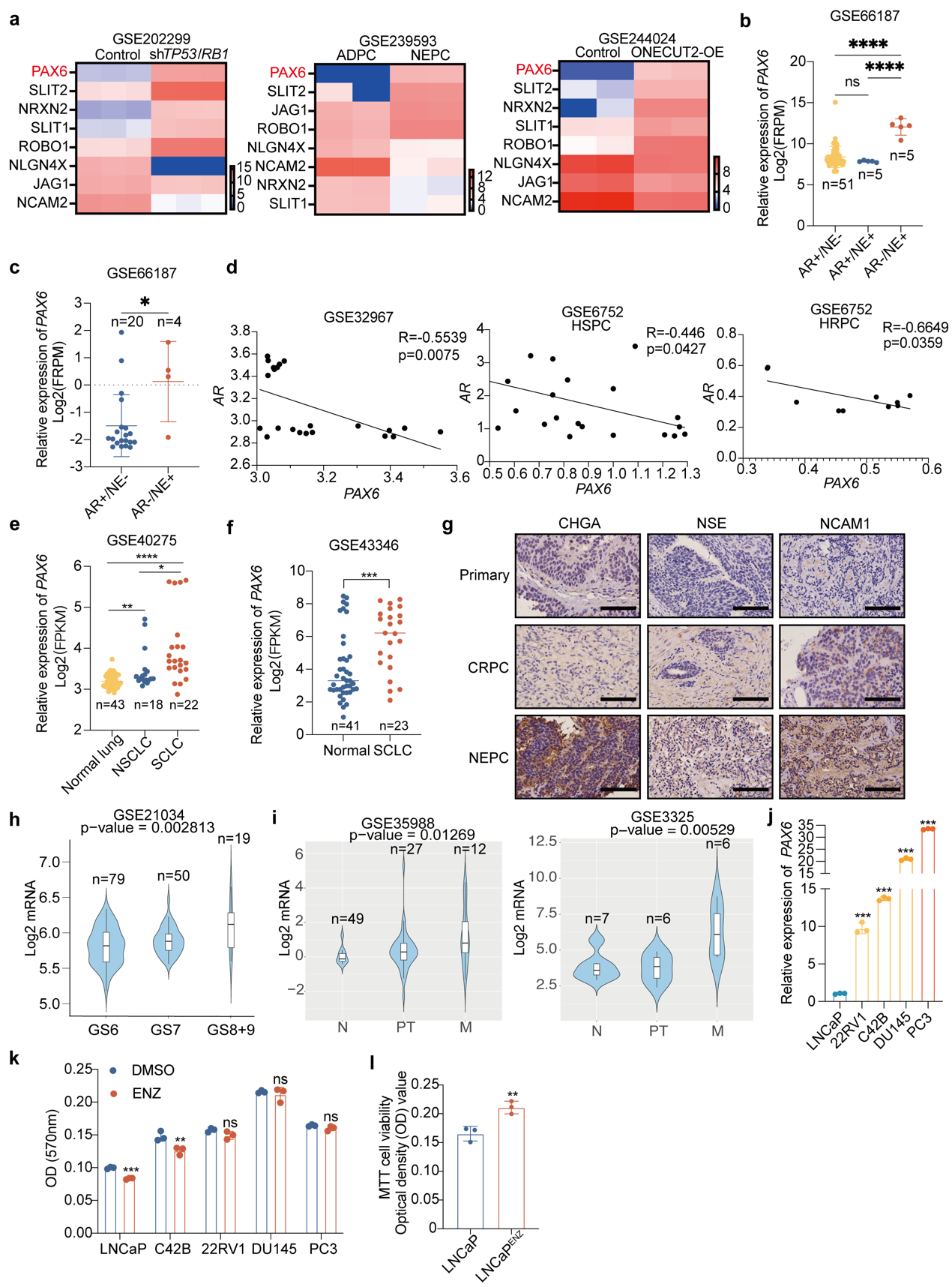


**Fig.S1** The expression of *PAX6* is upregulated in tumor cells with NE trans-differentiation. **a** Heatmap showing gene expression level of the neuron-related genes among the differentially expressed genes common to GSE202299, GSE239593 and GSE244024 datasets**. b** Comparisons of *PAX6* mRNA levels in samples from AR^+^/NE^-^(n=51), AR^+^/NE^+^ (n=5) and AR^-^/NE^+^ (n=5) patient samples based on GSE66187 dataset. **c** Comparisons of *PAX6* mRNA levels in AR^+^/NE^-^ (n=20) and AR^-^/NE^+^ (n=4) mouse samples based on GSE66187 dataset. **d** Correlation analysis of the expression of *PAX6* and *AR* based on GSE32967 and GSE6752 datasets. **e** Comparisons of *PAX6* mRNA levels in normal samples (n=43), NSCLC samples (n=18) and SCLC samples (n=22) based on GSE40275 dataset. **f** Comparisons of *PAX6* mRNA levels in normal samples (n=41) and SCLC samples (n=23) based on GSE43346 dataset. **g** Representative IHC staining of CHGA, NSE and NCAM1 in tissues from patients with Primary PCa, CRPC or NEPC (Scale Bar: 100 µm). **h** Quantification of *PAX6* mRNA levels in patients with different gleason scores (GS) based on GSE21034 dataset. **i** **﻿**Quantification of *PAX6* mRNA levels in normal adjacent prostate tissue (N), prostate cancer tissue in situ (PT) and metastatic prostate cancer tissue (M) in GSE35988 and GSE3325 datasets. **j** mRNA expression level of *PAX6* in PCa cell lines. **k** MTT cell proliferation assay of different PCa cell lines under ENZ (20 µM) treatment. **l** MTT cell proliferation assay of LNCaP and LNCaP^ENZ^ cells. All the experiments were repeated for three times. Data represent the mean ± SD. **, p<0.01; ***p < 0.001.


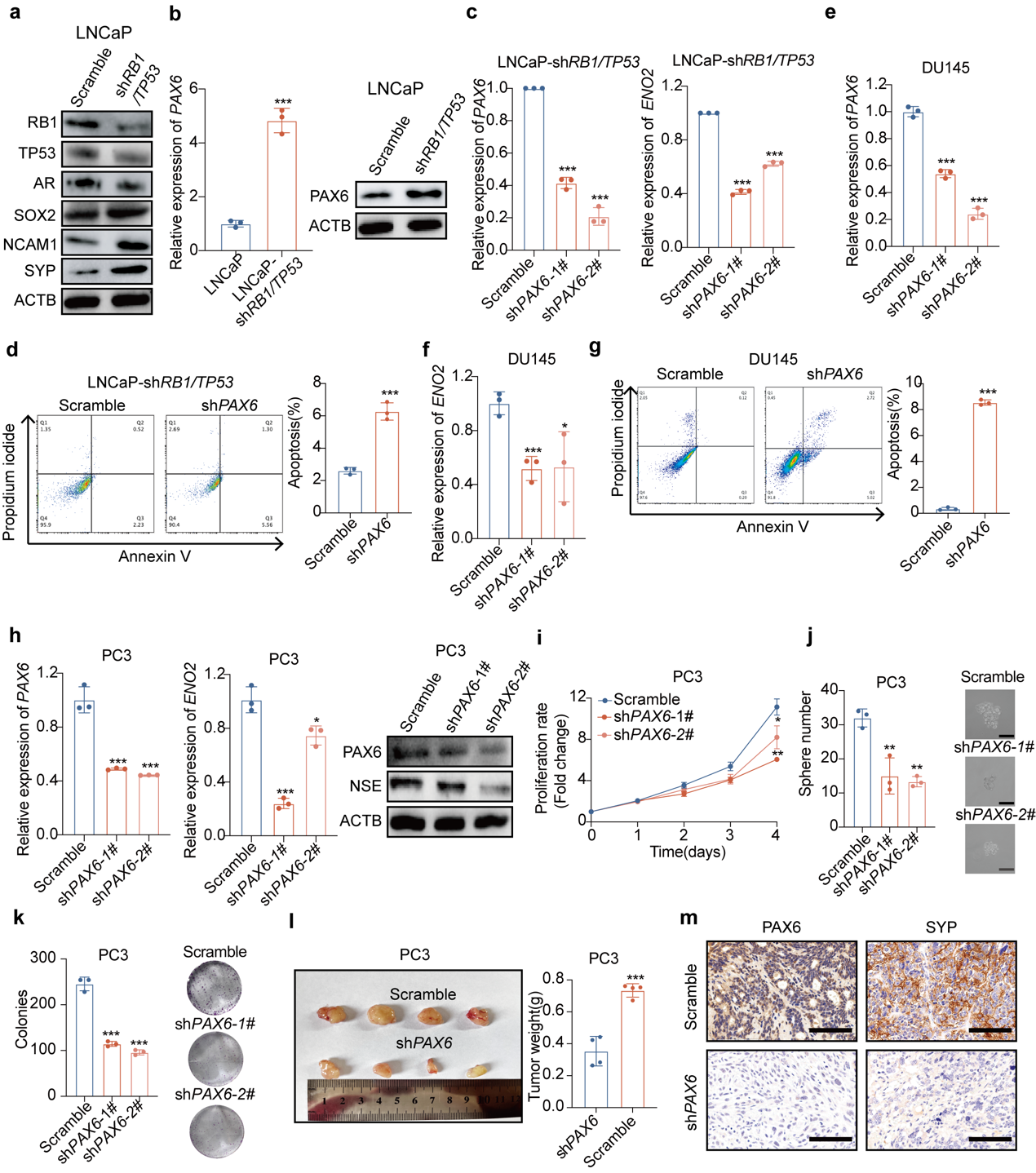


**Fig.S2** Knockdown of *PAX6* represses NE trans-differentiation in LNCaP-sh*RB1/TP53*, DU145 and PC3 cells. **a** Identification of the construction of LNCaP-sh*RB1/TP53* cells. **b** mRNA and protein expression of *PAX6* in LNCaP-sh*RB1/TP53* and the control cells. **c** mRNA expression of *PAX6* and *ENO2* in LNCaP-sh*RB1/TP53* and control cells after knockdown of *PAX6*. **d** Apoptosis assay in LNCaP-sh*RB1/TP53* cells after knockdown of *PAX6*. **e** mRNA expression of *PAX6* in DU145-sh*PAX6* and control cells. **f** mRNA expression of *ENO2* in DU145-sh*PAX6* and control cells. **g** Apoptosis assay in DU145-sh*PAX6* cells and control cells. **h** mRNA and protein expression of *PAX6* and *ENO2* in PC3-sh*PAX6* and control cells. **i** Cell proliferation assays in PC3-sh*PAX6* cells and control cells. Data represent the fold change of OD value during an observation period of up to 4 days. Fold change on the day of cell seeding (day0) in each group was set as 1. **j** Representative image and quantification assay of tumorsphere formation in PC3-sh*PAX6* cells and control cells*.* **k** Representative image and quantification assay of colony numbers in PC3-sh*PAX6* cells and control cells. **l** Anatomic tumor image of PC3-sh*PAX6* cells and control cells inoculated xenografts. **m** Representative IHC staining of PAX6 and SYP in xenograft samples (Scale Bar: 100 µm). All the experiments were repeated for three times. Data represent the mean ± SD. *, p<0.05; ***p < 0.001.


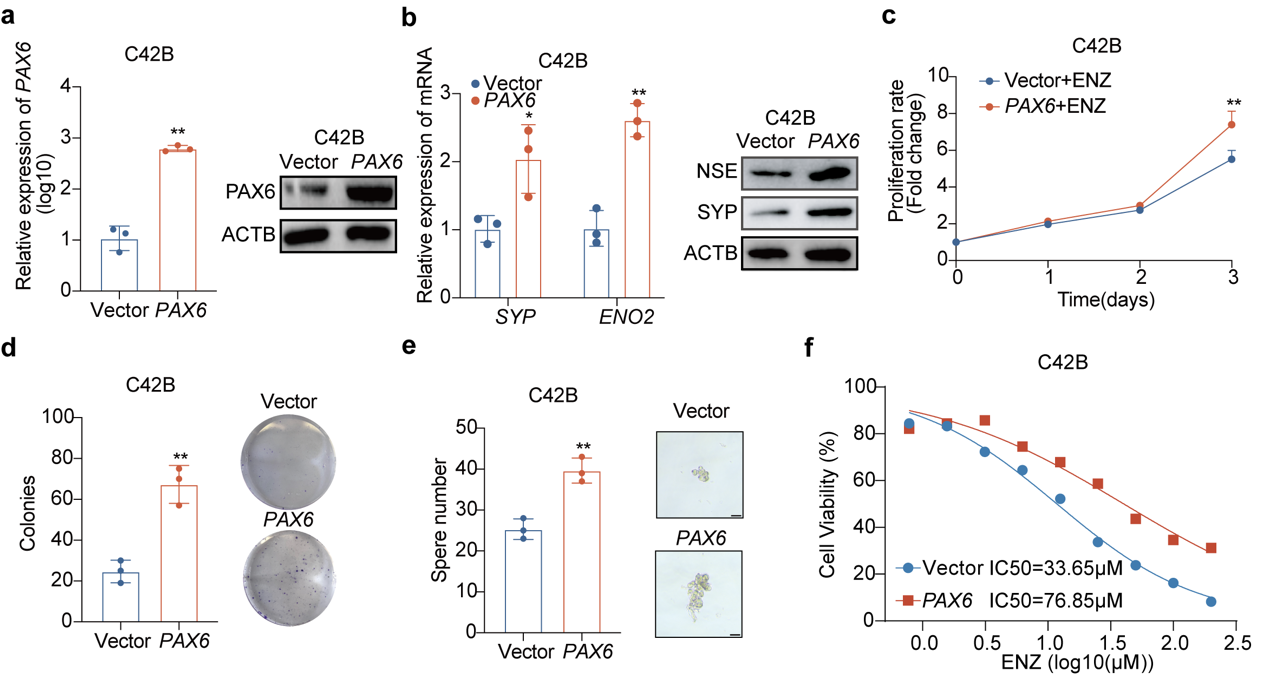


**Fig.S3** Over-expression of *PAX6* promotes the NE trans-differentiation in C42B cells. **a** mRNA and protein expression of *PAX6* in C42B-*PAX6* cells and control cells. **b** mRNA and protein expression of *SYP* and *ENO2* genes in C42B-*PAX6* cells and control cells. **c** Cell proliferation assays in C42B-*PAX6* cells and control cells. Cell proliferation assays were performed under the treatment of ENZ (20 µM). **d** Representative image and quantification assay of colony number in C42B-*PAX6* cells and control cells. **e** Representative image and quantification assay of tumorsphere formation in C42B-*PAX6* cells and control cells. **f** ENZ dose–response curves for C42B-*PAX6* cells and control cells. All the experiments were repeated for three times. Data represents the mean ± SD. *p < 0.05, **p < 0.01, ***p < 0.001.


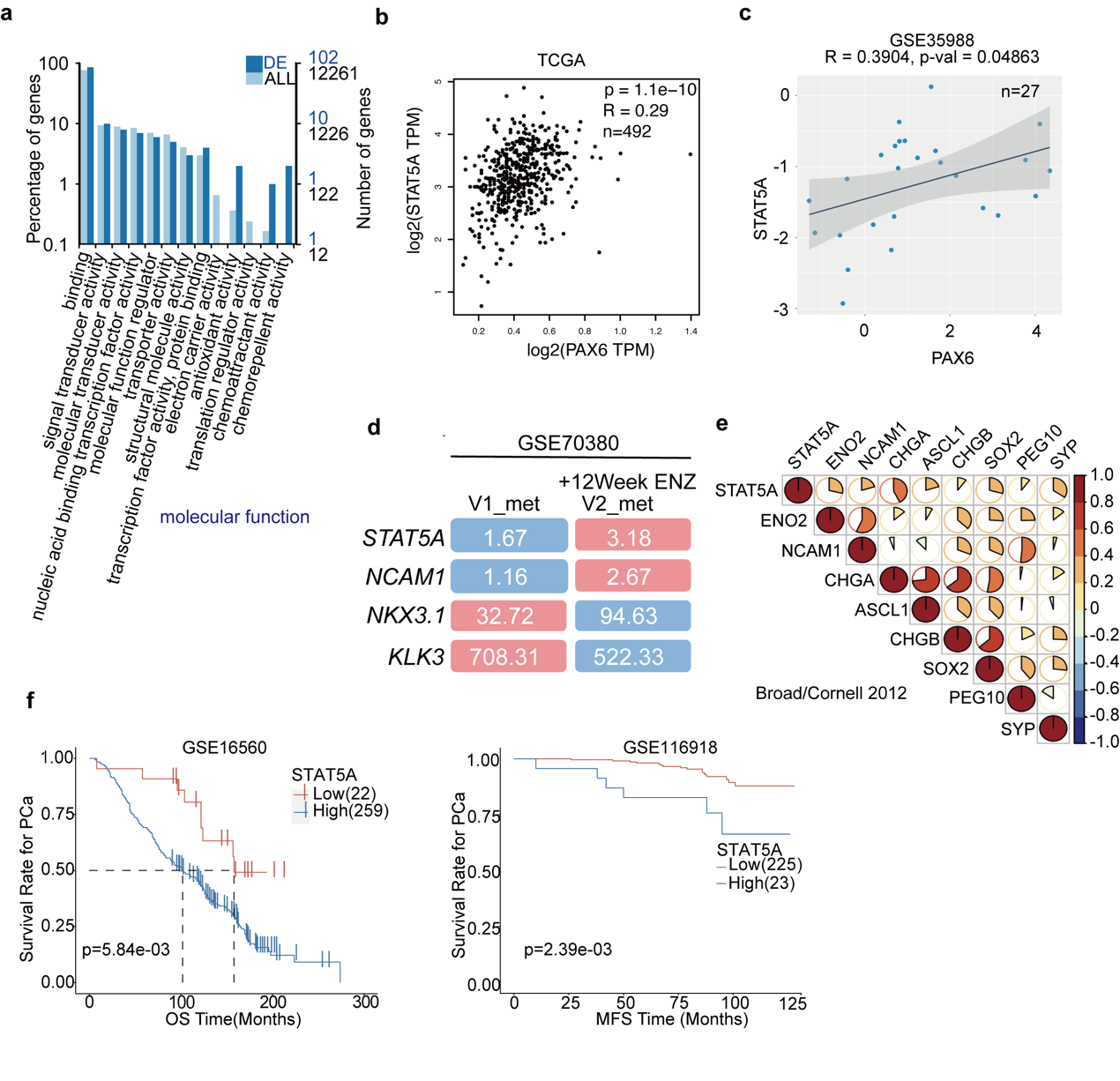


**Fig.S4** *PAX6* promotes the NE trans-differentiation via upregulating *STAT5A* expression. **a** GO analysis of RNA-seq results in DU145-sh*PAX6* cells and control cells. **b** Correlation analysis of *PAX6* and *STAT5A* expression based on the TCGA database (n=492). **c** Correlation analysis of *PAX6* and *STAT5A* expression based on the GSE35988 dataset (n=27). **d** Bioinformatics assay of the mRNA expression of *STAT5A, NCAM1, NKX3.1* and *KLK3* based on the GSE70380 dataset. **e** Correlation analysis of the expression of *STAT5A* and NE signature genes based on the Broad/Cornell 2012 Cohort. **f** Kaplan–Meier survival analysis of the relationship between *STAT5A* expression in tissues and the overall survival (OS) time based on the GSE16560 dataset and the metastasis-free survival (MFS) time based on the GSE116918 dataset.


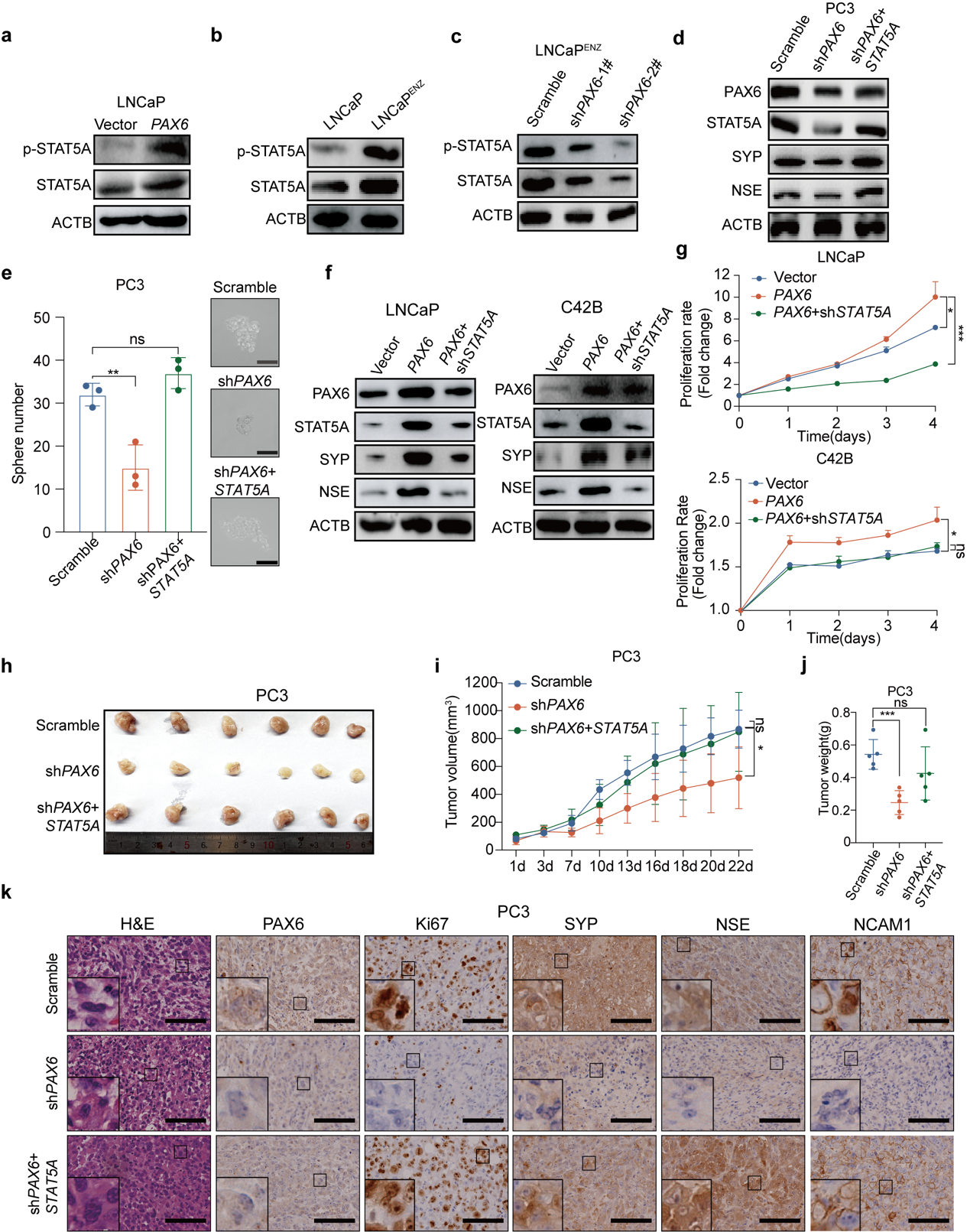


**Fig.S5** Elevated expression of *PAX6* promotes tumor growth and NE trans-differentiation via activation of the *PAX6/STAT5A* axis in vitro and in vivo. **a** Protein expression of STAT5A and p-STAT5A in LNCaP-*PAX6* cells. **b** Protein expression of p-STAT5A and STAT5A in LNCAP^ENZ^ and control cells. **c** Protein expression of STAT5A and NE markers p-STAT5A after *PAX6* knockdown in LNCaP^ENZ^ cells. **d** Protein expression of PAX6, STAT5A, SYP and NSE in PC3-sh*PAX6* cells with or without *STAT5A* overexpression. **e** Representative image and quantification assay of tumorsphere formation in PC3- sh*PAX6* cells with or without *STAT5A* overexpression. **f** Protein expression of PAX6, STAT5A, SYP and NSE in LNCaP-*PAX6* and C42B-*PAX6* cells with or without *STAT5A* knockdown. **g** Cell proliferation assay in LNCaP-*PAX6* and C42B-*PAX6* cells with or without *STAT5A* knockdown. **h** Anatomic tumor images and tumor weight analysis of PC3-sh*PAX6* cells inoculated xenografts with or without *STAT5A* overexpression (n=6). **i** Tumor volume analysis of PC3-Scramble, PC3-sh*PAX6* and PC3-sh*PAX6*+*STAT5A* cells inoculated xenografts respectively (n=6). **j** Tumor weights analysis of PC3-Scramble, PC3-sh*PAX6* and PC3-sh*PAX6*+*STAT5A* cells inoculated xenografts respectively (n=6). **k** Representative H&E staining and IHC staining of PAX6, Ki67, SYP, NSE, NCAM1 in PC3-Scramble, PC3-sh*PAX6* or PC3-sh*PAX6*+*STAT5A* cells inoculated xenograft samples (Scale Bar: 100 µm, with the boxed region enlarged and shown on the left). All the experiments were repeated for three times. Data represent the mean ± SD. *, p<0.05; **, p<0.01.


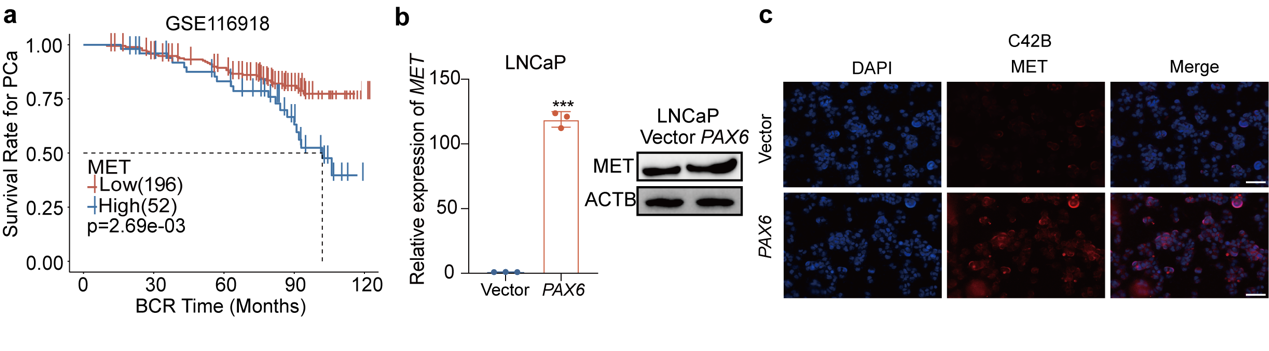


**Fig. S6** PAX6 regulates the expression of MET and high expression of MET is associated with worse prognosis. **a** Kaplan–Meier survival analysis of the relationship between *MET* expression in tissues and biochemical recurrence (BCR) time based on the GSE116918 dataset. **b** mRNA and protein expression level of MET in LNCaP-*PAX6* and control cells. **c** Protein expression level of MET in C42B-*PAX6* and control cells (Scale Bar: 100 µm).


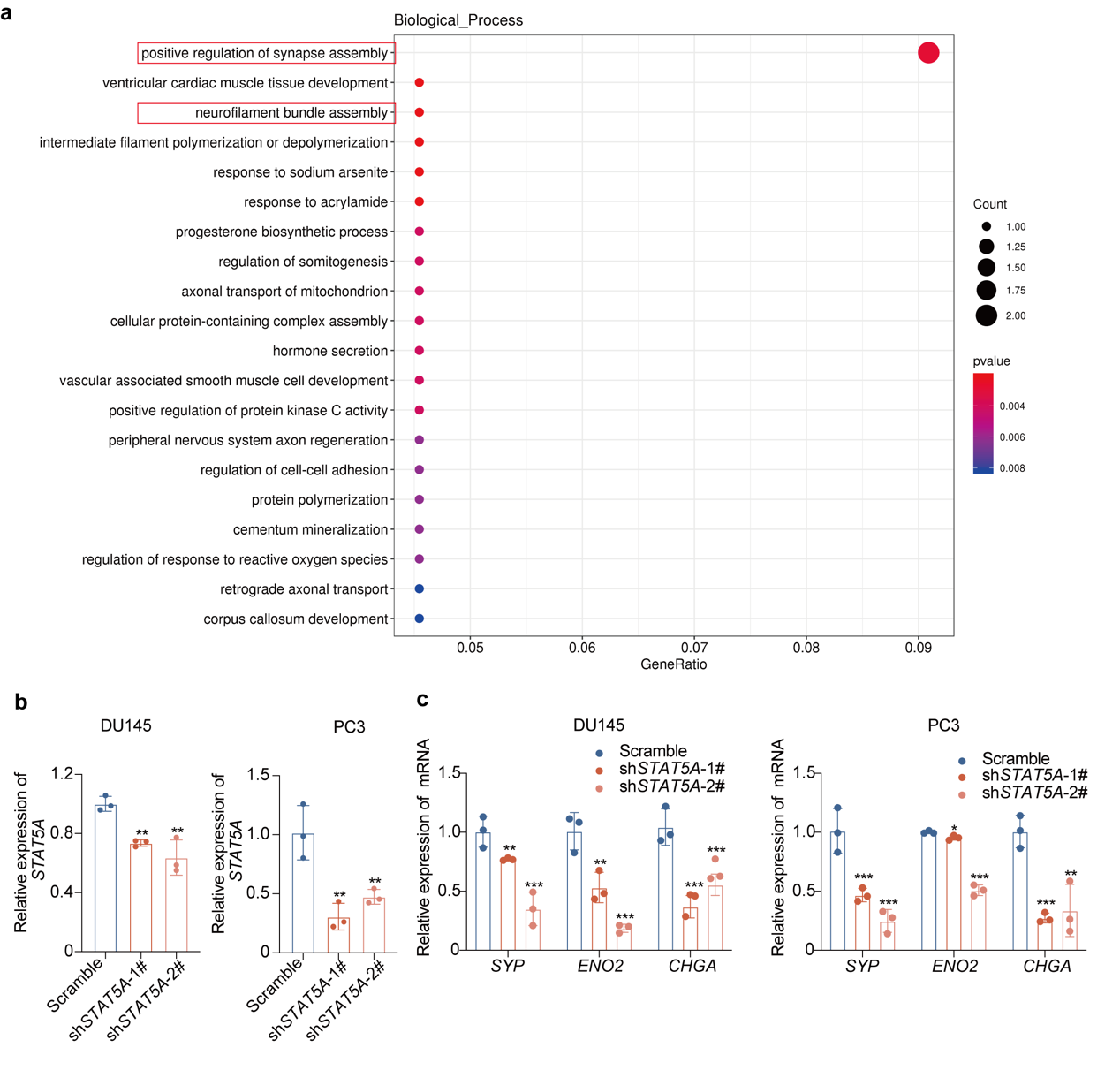


**Fig.S7** Knockdown of *PAX6* or *STAT5A* expression decreased the NE related pathways and genes. **a** Biological process analysis in DU145-sh*PAX6* cells vs. the control cells. **b** mRNA expression of *STAT5A* in DU145-sh*STAT5A* or PC3-sh*STAT5A* cells. **c** mRNA expression of *SYP, ENO2, CHGA* in DU145-sh*STAT5A* or PC3-sh*STAT5A* cells compared to that in the relevant control cells. All the experiments were repeated for three times. Data represent the mean ± SD. **, p<0.01; ***p < 0.001.
