# Supplementary material for: PAX6 promotes neuroendocrine phenotypes of prostate cancer via enhancing MET/STAT5A-mediated chromatin accessibility
Source: J Exp Clin Cancer Res. 2024 May 15;43:144. doi: 10.1186/s13046-024-03064-1 (PMC11094950; doi:10.1186/s13046-024-03064-1)
Supplement: Supplementary file 2 — Supplementary Material 2 [file 13046_2024_3064_MOESM2_ESM.docx]

**Supplementary Table1:** List of primers, sgRNA and shRNA sequences used in the study

**Cloning primers**

|  | **Forward** | **Reverse** |
| --- | --- | --- |
| PAX6-promoter-cloning primers | CCTAACTGGCCGGTACCATGCACAGCGGACTTGA | CCGGATTGCCAAGCTTGAAGCCTGACCTCTGTCATC |

**RT-qPCR-Primers**

| **Gene** | **Forward** | **Reverse** |
| --- | --- | --- |
| *ACTB* | *CACCATTGGCAATGAGCGGTTC* | *AGGTCTTTGCGGATGTCCACGT* |
| *PAX6* | CACACCGGTTTCCTCCTTCA | GGCAGAGCGCTGTAGGTGTT |
| *AR* | TACCAGCTCACCAAGCTCCT | GCTTCACTGGGTGTGGAAA |
| *KLK3* | CGCAAGTTCACCCTCAGAAGGT | GACGTGATACCTTGAAGCACACC |
| *ALDA1A3* | CTGCTACAACGCCCTCTATGCA | GTCGCCAAGTTTGATGGTGACAG |
| *STEAP4* | AGTCAGGAGCACTGGATGCAAG | CTTTGGCTGCCATGAGTGATCC |
| *PMEAP1* | CTGAGCCACTACAAGCTGTCTG | GGATTCCGTTGCCTGACACTGT |
| *NKX3.1* | CGCAGAACGACCAGCTGAGCA | CCTGAAGTGTTTTCAGAGTCCAAC |
| *ENO2* | GAACTATCCTGTGGTCTCC | CGACATTGGCTGTGAACTTG |
| *SYP* | TCAGTTCCGGGTGGTCAAG | AAGACCCATTGCAGCACCTT |
| *CHGA* | GGTTCTTGAGAACCAGAGCAGC | GCTTCACCACTTTTCTCTGCCTC |
| *NCAM1* | CATCACCTGGAGGACTTCTACC | CAGTGTACTGGATGCTCTTCAGG |
| *STAT5A* | CGGTTTGAGTGAGGGTTT CT | GTGGGCAACAGCATCATAGA |
| *MET* | TGCACAGTTGGTCCTGCCATGA | CAGCCATAGGACCGTATTTCGG |
| *KMT5C* | ACGAGGTGACATGCTTCTACGG | CTGGTTCGGAAAGCTCCTTCAC |
| *SMYD5* | CCTCTATGAGGAAGCAGTCAGC | ACTCCAGAGTGTCACAGGCATG |

**shRNAs and sgRNAs sequence**

|  | **Forward** | **Reverse** |
| --- | --- | --- |
| sgMET | CACCGCATCAGAACCAGAGGCTTGGT | AAACACCAAGCCTCTGGTTCTGATGC |
| shSTAT5A#1 | CACCGTCCGGCACATTCTGTACAATG CTCGAGCATTGTACAGAATGTGCCGGA TTTTTG | AAACCAAAAATCCGGCACATTCTGTACAATG CTCGAGCATTGTACAGAATGTGCCGGA C |
| shSTAT5A#2 | CACCG CCCACGTTTCCCGGGATATAT CTCGAGATATATCCCGGGAAACGTGGG TTTTTG | AAACCAAAAACCCACGTTTCCCGGGATATAT CTCGAG ATATATCCCGGGAAACGTGGG C |
| shAR#1 | CACCGCACCAATGTCAACTCCAGGATCTCGAGATCCTGGAGTTGACATTGGTGTTTTTG | AAACCAAAAACACCAATGTCAACTCCAGGATCTCGAGATCCTGGAGTTGACATTGGTGC |
| shAR#2 | CACCGCCTGCTAATCAAGTCACACATCTCGAGATGTGTGACTTGATTAGCAGGTTTTTG | AAACCAAAAACCTGCTAATCAAGTCACACATCTCGAGATGTGTGACTTGATTAGCAGGC |
| sgAR#1 | CACCGAGCAGCAAGAGACTAGCCCC | AAACGGGGCTAGTCTCTTGCTGCTC |
| sgAR#2 | CACCGCGGCTTAAGCAGCTGCTCCG | AAACCGGAGCAGCTGCTTAAGCCGC |
| sgAR#3 | CACCGCCTCGGTAGGTCTTGGACGG | AAACCCGTCCAAGACCTACCGAGGC |
| sgAR#4 | CACCGTCTCCCCAAGCCCATCGTAG | AAACCTACGATGGGCTTGGGGAGAC |

|  |
| --- |

**ChIP-qPCR Primers**

|  | **Forward** | **Reverse** |
| --- | --- | --- |
| PAX6-promoter | TTGCAGCGTCCTCACTGTC | AATAGGGCTGAAGAGCTGAGG |
| MET promoter | TTAGCGGAGACGTGGGAGAG | CCCACGACAAGGTGAAACTTTC |
| STAT5A-promoter-1 | CAGATGGAGACCTAAGGAGAC | CCACCACTCCTGGCTAATT |
| STAT5A-promoter-2 | ATCAGGTGCCCAGATGTCAGT | TGTCGGTGGCTGGATACAAA |
| AR-promoter-1 | GCGATTGATGAGTCTAGAGTC | GATGGGTTGTAGCTTTGTTGTAGC |
