# Supplementary material for: PAX6 promotes neuroendocrine phenotypes of prostate cancer via enhancing MET/STAT5A-mediated chromatin accessibility
Source: J Exp Clin Cancer Res. 2024 May 15;43:144. doi: 10.1186/s13046-024-03064-1 (PMC11094950; doi:10.1186/s13046-024-03064-1)
Supplement: Supplementary file 6 — Supplementary Material 6 [file 13046_2024_3064_MOESM6_ESM.docx]

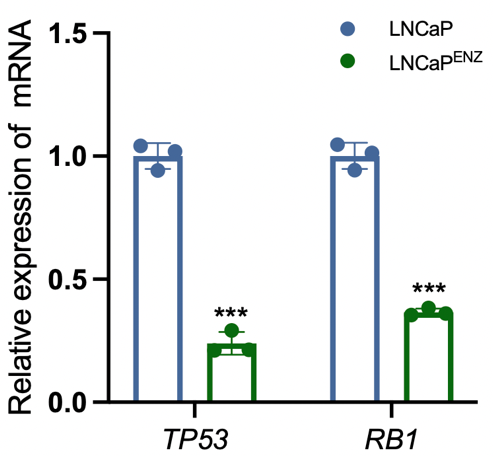
**Response Fig. 1** The mRNA expression level of *TP53* and *RB1* was downregulated in LNCaP^ENZ^
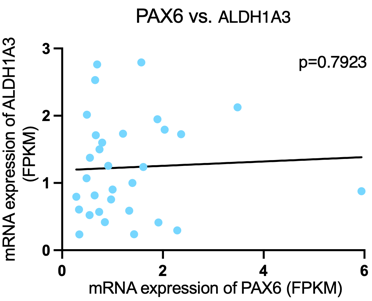

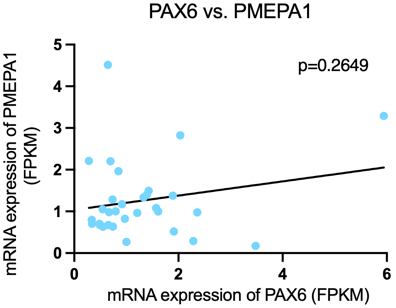

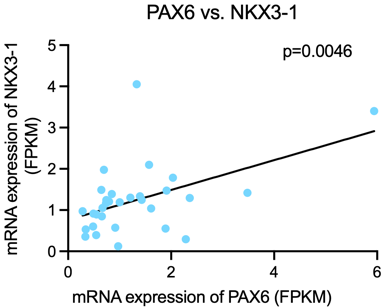
cell compared to control.

**Response Fig. 2** Correlation analysis of PAX6 with AR associated genes based on the Broad/Cornell 2012 cohort.
