# Supplementary figures and images for: PAX6 promotes neuroendocrine phenotypes of prostate cancer via enhancing MET/STAT5A-mediated chromatin accessibility
Source: J Exp Clin Cancer Res. 2024 May 15;43:144. doi: 10.1186/s13046-024-03064-1 (PMC11094950; doi:10.1186/s13046-024-03064-1)

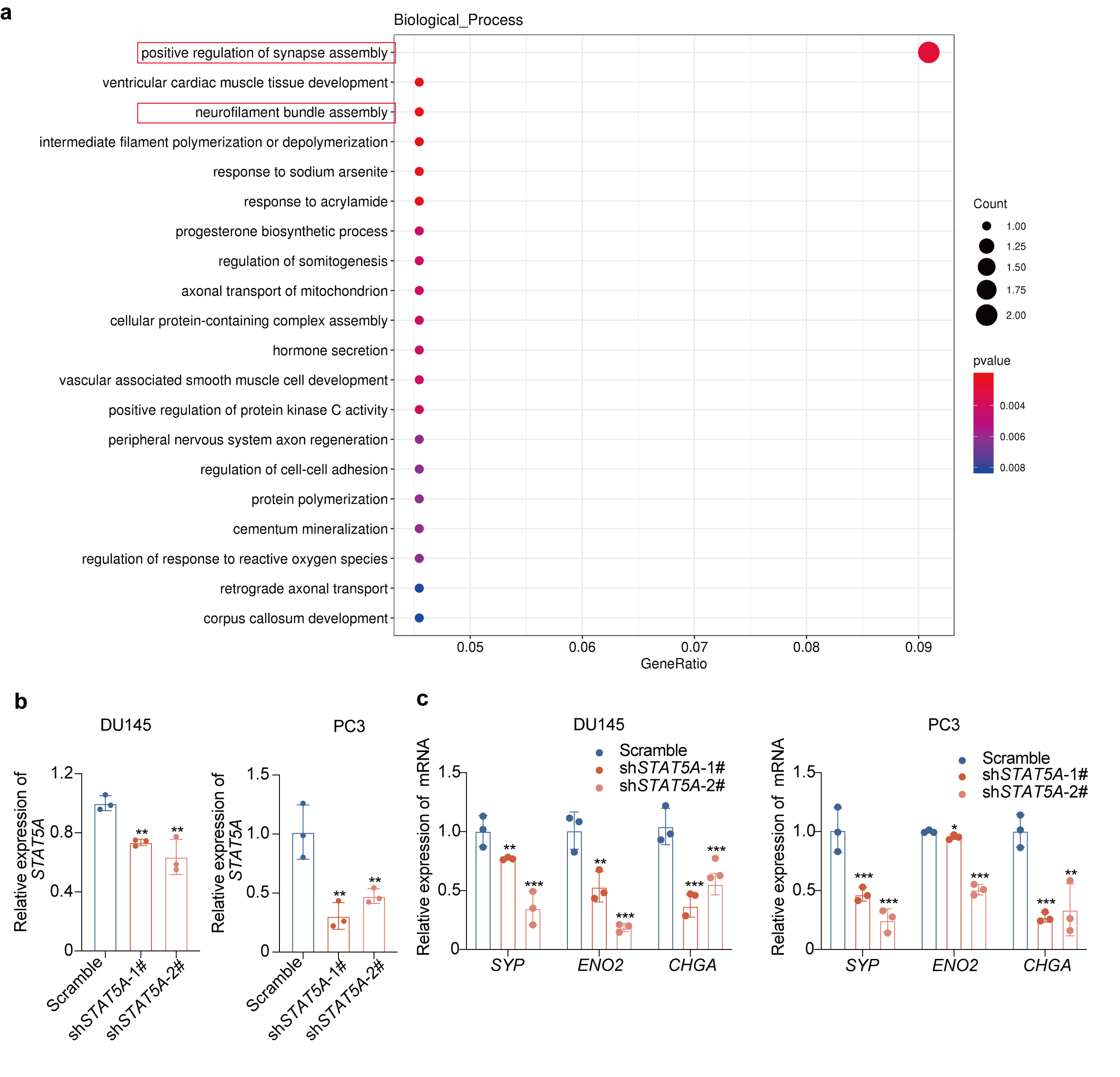

Supplement: Supplementary file 7 — Supplementary Material 7 [file 13046_2024_3064_MOESM7_ESM.png]
